# Gut microbes and food reward: From the gut to the brain

**DOI:** 10.3389/fnins.2022.947240

**Published:** 2022-07-25

**Authors:** Alice de Wouters d’Oplinter, Sabrina J. P. Huwart, Patrice D. Cani, Amandine Everard

**Affiliations:** Metabolism and Nutrition Research Group, Louvain Drug Research Institute (LDRI), Walloon Excellence in Life Sciences and BIOtechnology (WELBIO), UCLouvain, Université catholique de Louvain, Brussels, Belgium

**Keywords:** food reward, food intake, gut microbes, gut microbiome, gut-brain-axis, obesity

## Abstract

Inappropriate food intake behavior is one of the main drivers for fat mass development leading to obesity. Importantly the gut microbiota-mediated signals have emerged as key actors regulating food intake acting mainly on the hypothalamus, and thereby controlling hunger or satiety/satiation feelings. However, food intake is also controlled by the hedonic and reward systems leading to food intake based on pleasure (i.e., non-homeostatic control of food intake). This review focus on both the homeostatic and the non-homeostatic controls of food intake and the implication of the gut microbiota on the control of these systems. The gut-brain axis is involved in the communications between the gut microbes and the brain to modulate host food intake behaviors through systemic and nervous pathways. Therefore, here we describe several mediators of the gut-brain axis including gastrointestinal hormones, neurotransmitters, bioactive lipids as well as bacterial metabolites and compounds. The modulation of gut-brain axis by gut microbes is deeply addressed in the context of host food intake with a specific focus on hedonic feeding. Finally, we also discuss possible gut microbiota-based therapeutic approaches that could lead to potential clinical applications to restore food reward alterations. Therapeutic applications to tackle these dysregulations is of utmost importance since most of the available solutions to treat obesity present low success rate.

## Gut-brain axis related to food intake

The gut-brain axis is a complex bi-directional communication system connecting the gastrointestinal (GI) tract and the central nervous system (CNS). This connection allows the brain to be informed among other components of the energy status in the periphery. The CNS sends then feedbacks to maintain energy homeostasis (Cryan et al., 2019). Two pathways are involved in this communication: the nervous and the systemic pathways.

### Nervous pathway

The GI tract is the largest place where intestinal neurons are following in number those from the CNS (i.e., often seen as our second brain) and clearly connected to the brain. Afferent fibers convey sensory information from the upper gastrointestinal tract to the CNS *via* vagal and splanchnic nerve pathways. Gut vagal and splanchnic afferents play a role in the control of satiation (Sclafani et al., 2003). These connections maintain the brain informed about the energy status from the periphery. The most afferences described in the control of food intake are vagal afferences. These vagal afferences come from the intestine and have their cell bodies in the nodose ganglia and project to the nucleus tractus solitarus (NTS) in the brainstem. The NTS contains projections to several regions of the brain including the hypothalamus (Berthoud et al., 2004; Rinaman, 2010). Vagal afferents detect mobility or distension and express a vast variety of GI hormones receptors such as glucagon-like peptide-1 (GLP-1), cholecystokinin (CCK), peptide YY (PYY) or ghrelin receptors (Berthoud et al., 2004; Ye and Liddle, 2017). It has been proven that specific stimuli from the GI tract activate distinct population of vagal afferents (Egerod et al., 2018; Wang et al., 2020).

Few years ago, functional synapses between enteroendocrine cells (EECs) and vagal afferents nerves have been discovered. Basolateral cytoplasmic elongations of EECs, called neuropods, connects directly to the vagal pathway permitting a fast and precise signal to the brain, through the nodose ganglia (Kaelberer et al., 2018; Liddle, 2019).

The key role of the vagus nerve in the gut-brain axis regulating the non-homeostatic food intake was shown by Han et al. (2018a). For the first time, the authors demonstrated using optogenetics tools a functional pathway linking the gut to the brain reward system, involving the vagus nerve, the right nodose ganglion, the NTS, and the dorsal striatum (Han et al., 2018a).

### Systemic pathway

**FIGURE 1:**
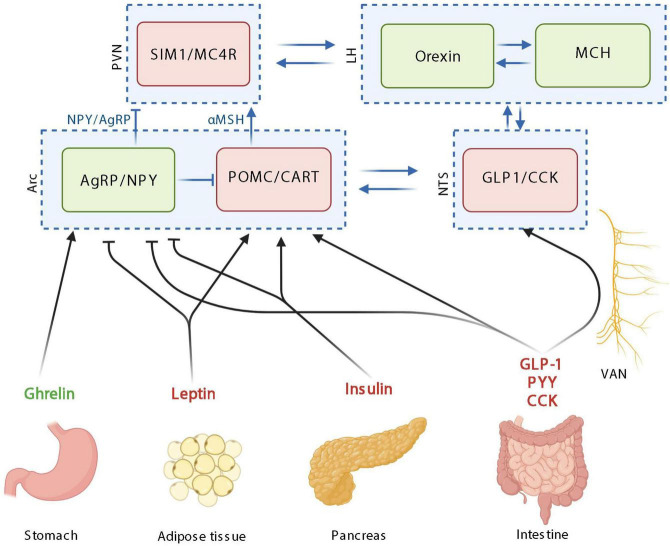
The homeostatic control of food intake upon peripheric signals. Orexigenic hormones or neurotransmitters are in green; anorexigenic hormones or neurotransmitters are in red. PVN: paraventricular nucleus; SIM1, single–minded family BHLH transcription factor 1; LH, lateral hypothalamus; MCH, melanin-concentrating hormone; Arc, arcuate nucleus; AgRP, agouti-related protein; NPY, neuropeptide Y; POMC, pro-opiomelanocortin; CART, cocaine-amphetamine-related transcript; NTS, nucleus tractus solitarus; VAN, vagal afferent nerves; CCK, cholecystokinin; GLP-1, glucagon-like peptide 1; PYY, peptide YY; MC4R, Melanocortin 4 receptor. Created with BioRender.com.

Besides neurons, the gut also contains various type of cells. The gut is also one the largest compartment where the immune system is developed [e.g., the gut-associated lymphoid tissue (GALT) and immune infiltrating cells]. Moreover, although the intestinal EECs are representing only 1% of the total intestinal epithelial cells, the gut constitutes the biggest endocrine organ (Plovier and Cani, 2017). Thirty hormones have been identified in the GI tract and therefore the intestine represents an incredible reservoir of peptides acting at distance from the gut and on different organs. EECs are present all along the GI tract and form an important endocrine organ implicated in the control of food intake as described in the previous paragraph. In a postprandial state, EECs secrete GLP-1, CCK, and PYY all of them acting as anorexigenic hormones (Latorre et al., 2016). In contrast, ghrelin is released by the stomach and acts as an orexigenic hormone (Wren et al., 2001). These hormones can be released in the blood or activates the vagal afferents nerves, thereby targeting the brain (Figure 1).

## Food intake control in physiology and pathology

Food intake is controlled according to energy expenditure to maintain a stable body weight over time through a process called homeostatic control of food intake (Morton et al., 2014). However, food intake is also controlled by a non-homeostatic system, namely the reward system. This system induces an eating behavior for pleasure rather than for energy needs and stimulates the intake of high nutritional value food, rich in sugar and/or fat, known as “palatable food” (Stice et al., 2013; Alastair et al., 2015). Even if some alterations of the homeostatic system of food intake have been reported during obesity and are still an important field of research, there is now a very strong current of thought that a major cause of the increase in food intake associated with the rise of obesity resides in the hedonic rather than the homeostatic system. Indeed, in the present context of the increased availability, accessibility and affordability of energy-dense foods also referred as the obesogenic environment, homeostatic system can be overridden by the hedonic control of food intake (Volkow et al., 2011). That said, the investigation of the homeostatic control of food intake is still important to consider.

### Homeostatic control of food intake

#### In physiology

Based on these peripheral signals relayed to the brain, food intake, appetite and satiety are mainly integrated at the level of hypothalamic neuronal circuits (Figure 1; Joly-Amado et al., 2014). Neurons within the hypothalamus play critical roles in the homeostatic control of energy and body weight by adjusting energy intake to energy expenditure in response to biological and environmental cues.

The homeostatic feeding is mainly orchestrated in the arcuate nucleus of the hypothalamus (Arc) by different types of neuron populations: the co-expressing Agouti-related protein (AgRP) and neuropeptide Y (NPY) neurons and the co-expressing pro-opiomelanocortin (POMC) and cocaine-amphetamine-related transcript (CART) neurons, with orexigenic and anorexigenic functions, respectively (Figure 1; Morton et al., 2006). Importantly, these populations of neurons interact together by inhibiting each other. Upon activation, POMC neurons release α-melanocyte-stimulating hormones (α-MSH), that binds to melanocortin 4 receptor (MC4R) in the paraventricular nucleus (PVN) and leads to decreased food intake. On the opposite, AgRP/NPY activation in the Arc, stimulates the release of AgRP and NPY, inhibiting MC4R-expressing neurons in the paraventricular nucleus of the hypothalamus (PVN). Overall, the PVN is characterized by neurons expressing the single minded 1 transcription factor (SIM1) (Figure 1; Morton et al., 2014). The Arc and PVN are located at a strategic position in the hypothalamus along the third ventricle. The third ventricle is more permeable due to its fenestred brain barrier, allowing peripheral signals to enter the hypothalamus and then bind receptors located in the Arc and PVN. Arc neurons senses metabolic signals from the periphery since it express receptors for circulating molecules, produced by peripheral organs such as PYY, ghrelin, leptin, and GLP-1 (Cone, 2005). The Arc communicates with the brainstem including the NTS, which also collects information from the periphery (Figure 1). The vagal afferent fibers end in the NTS, which also express gut peptides receptors including cholecystokinine receptor 1 (CCK1R) and GLP-1 receptor (GLP1R).

The main peripheral actors described in the homeostatic control of food intake are insulin, leptin ghrelin, CCK, GLP-1, PYY, and GIP (Figure 1). Insulin is produced and secreted by the pancreas into the circulation in response to postprandial increase in blood glucose and it readily reaches tissues throughout the body, including the brain. Even if the main effects of insulin in the periphery is to induce glucose uptake by the tissues, in the brain, the majority of glucose transport into neurons occurred independently of insulin. In the brain, insulin inhibits AgRP/NPY neurons and activates POMC neurons in the Arc thereby inducing anorexigenic effects (Figure 1; Bruning et al., 2000; Obici et al., 2002; Dodd and Tiganis, 2017).

The idea that fat mass signals are involved in the hypothalamic control of food intake has already been proposed in 1953 (Kennedy, 1953). Later, this signal from the fat mass has been identified with the discovery of leptin in 1994 (Zhang et al., 1994). Leptin is produced by adipocytes in a proportional quantity to the fat mass (Considine et al., 1996) and is released in the circulation thereby reaching the brain. Like insulin, leptin in the brain inhibits AgRP/NPY neurons and activates POMC neurons in the Arc inducing anorexigenic effects (Figure 1; Thornton et al., 1997; Wilson et al., 1999; Cowley et al., 2001; Varela and Horvath, 2012).

On the opposite, the main hormone involved in orexigenic effects is ghrelin. Ghrelin is synthetized by the stomach increases synthesis of AgRP and NPY in the Arc, thereby inducing its orexigenic effects (Figure 1; Kamegai et al., 2000; Tschop et al., 2000; Asakawa et al., 2001). Ghrelin also inhibits POMC neuronal activity, however, since ghrelin receptor expression has not been reported in POMC neurons, this inhibitory effect may be mediated by the activation of NPY/AgRP neurons interacting and inhibiting POMC neurons (Figure 1; Willesen et al., 1999; Chen et al., 2017).

Besides the stomach other parts of the intestine also synthetize peptides targeting the hypothalamus to control food intake. Enteroendocrine cells (EECs) located all along the intestine, from the duodenum to the colon, are involved in the synthesis and release of gastro-intestinal peptides some of which are involved in the homeostatic control of food intake.

CCK is produced by CCK cells, which are EECs located all along the intestine with the majority of them being in the duodenum (Polak et al., 1975; Egerod et al., 2012). CCK is produced in response to nutrients in the intestinal lumen, mainly lipids and protein and is inducing anorexigenic effect mainly by the activation of POMC neurons expressed in cells of the nucleus of the solitary tract, through the vagus nerve (Figure 1; Lorenz and Goldman, 1982; Liou et al., 2011; Wang et al., 2011; Warrilow et al., 2022).

The glucose-dependent insulinotropic peptide (GIP) is synthesized within and released from intestine, mainly in the duodenum and proximal jejunum (Mortensen et al., 2003). GIP is secreted in response to nutrient ingestion, especially glucose or fat and its effect on food intake are mainly mediated by GLP-1 (Roberge and Brubaker, 1993). GLP-1 is another anorexigenic peptide produced by EECs (L-cells) present at high density in the ileum and distal colon (Hansen et al., 2013). GLP-1 production and secretion is induced in response to the presence of nutrients in the intestine either by a direct or indirect effect mediated by the GIP (Roberge and Brubaker, 1993). Even if the activation of central GLP-1 receptors plays a role in mediating food intake effect of GLP-1, since the short half-life of GLP-1, it is likely that endogenous gut-derived GLP-1 suppresses food intake by acting through the vagus nerve (Abbott et al., 2005; Hayes et al., 2011). Both central and peripheral GLP-1 agonists are able to inhibit NPY and AgRP neurons and activate POMC neurons thereby inducing its anorexigenic effects (Figure 1; Seo et al., 2008; Secher et al., 2014; He et al., 2019). PYY is an additional anorexigenic peptide mainly secreted in ileum and large intestine EECs in response to fat ingestion (Morley et al., 1985; Bottcher et al., 1993). PYY induced its anorexigenic effects through the inhibition of NPY/AgRP neurons in the hypothalamus (Figure 1; Acuna-Goycolea and van den Pol, 2005). Besides the intestine, other organs also contribute to the homeostatic control of food intake by secreting other hormones such as the adiponectin from the adipose tissue or amylin, polypeptide Y from the pancreas.

Importantly, the different hormones and brain structures do not act separately but interact to influence food intake (Ronveaux et al., 2015). In the next section (“Systemic pathway”), we will describe how reward-related structures influence food intake. The connections between the homeostatic and non-homeostatic controls of food intake are essential to maintain a stable weight and mainly occur at the level of the lateral hypothalamus (LH) (Figure 1). The LH is composed of 2 neuronal populations: one expressing melanin-concentrating hormone (MCH) and one expressing orexins. MCH neurons project to the nucleus accumbens (Nac), whereas orexin neurons project to the ventral tegmental area (VTA), two brain areas involved in the reward system (Murray et al., 2014).

Moreover, it is important to note that besides the control of food intake by acting on the brain (*via* endocrine and nervous routes), the main functions exerted by the gut peptides secreted are also linked to gastric emptying and intestinal motility, the regulation of secretion from the pancreas and the gallbladder and the stimulation of insulin secretion, also known as the incretin effect. Overall, gastric emptying and intestinal motility are also additional key players controlling satiation (Cummings and Overduin, 2007).

#### In physiopathology (obesity)

Over the past 25 years, sequencing studies have led to the discovery of several genes involved in homeostatic control of food intake whose disruption causes morbid obesity in humans. The detailed study of people with these monogenic obesity syndromes has provided unique insights into the role these neural circuits play in modulating human appetite and body weight (Farooqi, 2022). To date, almost all of these genes encode proteins involved in the leptin-melanocortin pathway (van der Klaauw and Farooqi, 2015). However, mutations that disrupt the production or bioactivity of leptin or in the gene encoding the leptin receptor are very rare and contribute to only 1–5% of severe obesity in children (Montague et al., 1997; Clement et al., 1998; Albuquerque et al., 2017). Therefore, other mechanisms are involved in inappropriate food intake during obesity.

In fact, hyperleptinemia and resistance to reducing body mass are two characteristics of typical obesity. Indeed, leptin is overexpressed at the gene level in the adipose tissue of individuals with obesity and strong positive associations exist between plasma leptin levels and body fat percentage (Lonnqvist et al., 1995; Considine et al., 1996). Therefore, several studies point toward leptin resistance instead of loss of leptin during obesity (Frederich et al., 1995). Similar observations are made for insulin since it is becoming increasingly apparent that neurons in the brain also become resistant to insulin; however, the relative contributions of central insulin resistance to the development of obesity and T2D remain poorly understood (De Souza et al., 2005; Dodd and Tiganis, 2017). In conclusion, any disruption in the production and or action of these mediators involved in homeostatic control of food intake can lead to overconsumption and obesity.

### Non-homeostatic control of food intake

#### In physiology

**FIGURE 2:**
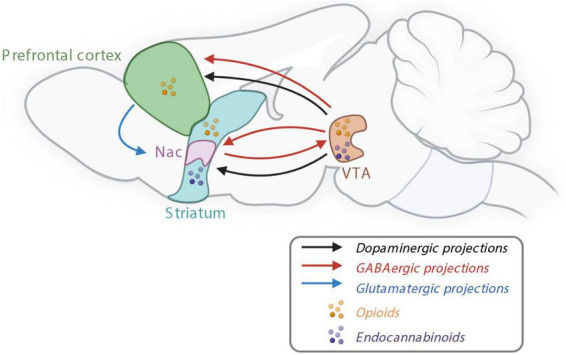
The reward system controlling non-homeostatic food intake. Nac, nucleus accumbens; GABA, γ-aminobutyric acid; VTA, ventral tegmental area. Created with BioRender.com.

As mentioned earlier, food intake is also influenced by non-homeostatic signals, inducing food intake for pleasure. The hedonic intake of food depends mainly on taste, odor, texture, and appearance (Rosenstein and Oster, 1988; Sorokowska et al., 2017; Tan et al., 2020). The reward system was essential for our hunter-gatherer ancestors to favor a good energy storage in order to increase the survival rate. However, today in most western countries, humans are in omnipresence of palatable food and do not have to face a period of famine. Therefore, in these conditions, the reward system can lead to overeating and positive energy balance and induce obesity (Wiss et al., 2018; de Araujo et al., 2020). In 1954, Olds and Milner were the first to identify rewarding sites in rat brains (Olds and Milner, 1954). Few years after this discovery, the catecholaminergic system has been highlighted to be implicated in the processes of reward (Poschel and Ninteman, 1963; Wise, 1977; Olds and Fobes, 1981). Nowadays it is well established that the reward system involved the mesocorticolimbic pathway that includes dopaminergic neurons located in the VTA and their axons projecting to the striatum, including the Nac and to the prefrontal cortex (PFC) (Figure 2; Arias-Carrion et al., 2010). Other structures have been proved to be implicated in reward processes such as amygdala, ventral pallidum and the hippocampus (Palmiter, 2007; Arias-Carrion et al., 2010; Kenny, 2011).

Dopamine seems to be the major and common driver of the food and drug reward in the mesocorticolimbic pathway (Robinson and Berridge, 2003; Garcia-Cabrerizo et al., 2021). It has already been demonstrated in 1989 that blocking the dopaminergic pathway inhibits the response for food during an operant task (Wise and Rompre, 1989). In response to rewarding stimuli, such as palatable food, dopamine is released in the mesocorticolimbic pathway promoting reward behaviors. In physiological state, dopamine is synthetized in two steps: first tyrosine is produced by a rate-limiting enzyme, the tyrosine hydroxylase (TH) to produce dihydroxyphenylalanine (DOPA), then the aromatic L-amino acid decarboxylase dopamine produces dopamine. Dopamine is stored in vesicles, before being released in the synaptic space. Eighty percent of the dopamine is recaptured by the dopamine transporter (DAT), whereas the rest is degraded into 3-methoxytyramine and 3,4-dihydroxyphenylacetic acid (DOPAC). Both are transformed into homovanillic acid (HVA).

In addition to dopaminergic neurons, γ-aminobutyric acid (GABA)-ergic and glutamatergic releasing neurons are also present in the VTA and involved in the reward processes—either by modulating the activity of dopamine neurons or independently of dopamine by sending projections to the brain structures innervated by VTA dopamine neurons (Figure 2; Morales and Margolis, 2017). Opioid signaling is also involved in the reward system since opioid receptors and their endogenous ligands are expressed in several areas of the brain, including in the VTA, Nac and PFC (Figure 2; Le Merrer et al., 2009). Bioactive lipids such as endocannabinoids, are involved in both homeostatic and rewarding mechanisms of food intake. Endocannabinoids, endocannabinoid receptors including CB1 receptors, and synthetizing enzymes are all present in the mesolimbic system and, in particular, in the Nac and VTA (Figure 2; Di Marzo et al., 2009).

The reward system encodes for the three psychological components of food intake: wanting, liking and learning. The dopaminergic system seems to control mostly the wanting component while GABAergic and opioid systems seem to be also implicated in the liking component (Robinson and Berridge, 2003; Garcia-Cabrerizo et al., 2021).

##### Wanting

The wanting component is related to the motivation to obtain a reward, and is the predominant behavior during the first phase of the food intake, the appetitive phase. This component induces an effort to acquire a specific nutrient in order to access to the rewarding value of it. A neuronal activation in the reward-related areas is observed in humans in response to visual cues of high caloric food compared to control food (Page et al., 2011). This component present dynamic fluctuations depending on the physiological state, when hungry, the “wanting” is significantly amplified (Berthoud et al., 2017).

In humans, the motivational component can be evaluated with analysis through a work to access rewarding food for example through computer game (Griffioen-Roose et al., 2010). While in rodents, the wanting component is often assessed with an operant conditioning test. The mouse must press on a lever to obtain a palatable food or other rewarding nutrients. The more the mouse is motivated, the more it will press on the lever. A principle of progressive ratio is often used to really assess the motivation with an incremental number of lever presses asked for each additional reward delivered, in contrast to the fixed ratio which delivers one reward after one lever press (Buccafusco, 2009).

##### Liking

The liking component refers to the hedonic part of the reward system; it is related to the pleasure felt by eating a specific food, prominent during the consummatory phase. Hedonic word derives from the ancient Greek *hedonikós*, meaning “pleasurable.” Before consumption of food, the hedonic component is neutral and become active only after ingestion (Robinson and Berridge, 1993, 2003).

In human adults, a subjective self-report is used to report the rate how much they like (or dislike) a stimulus (Thomsen, 2015). In animals and in young humans, orofacial reaction can be analyzed after an oral administration of substance to assess the liking component (Thomsen, 2015). This hedonic component is essential to discriminate control (neutral) food to palatable food and induces a preference for the intake of this latter: it helps to make a choice between tastes. The liking component can be assessed in animal models with a food preference test. The preference is analyzed using two or more food samples free access presenting to animals. In humans, lists of ranked food are often asked (de Araujo et al., 2020).

##### Learning

An attraction to certain type of food like sweet exists due to a conditioning process. It is a learning process making the link between associated stimuli and the pleasure felt during post-absorption *via* a Pavlovian conditioning. This component of the food reward occurs after the ingestion, and will strongly influence the wanting, depending on the type of liking experienced. In his experiment, Pavlov rang a bell every time he served food to his dog. Then 1 day, Pavlov rang the bell without giving food at its dog. However, this one was already drooling. It was the discovery of the learning process (Clark, 2018). The brain anticipates the rewarding value of a specific food and encourages its absorption. This component is essential to make the link between stimuli and long-term effects on health. This component is stable over the time and also helps to make choice between different food (Kringelbach et al., 2012). For example, the gastro-intestinal consequences of a food intake will impact the future intake of this type of food.

With rodents, the learning component can be assessed with a conditioned place preference. The system is made of two different easily recognizable compartments characterized by different walls and grounds. To know the preferred compartment in baseline, the time spent by the mouse in each compartment is recorded during a pre-test with full access to each compartment. During trainings, the mouse is restrained in one compartment with the associated treatment: the less preferred compartment is used for rewarding trainings, the mouse can have access to palatable food whereas the most preferred compartment is used as control. After several days of trainings, the test ends with a session of free access to each compartment in absence of food. The aim of this test is to reverse the less preferred compartment due to food rewards received during trainings (Buccafusco, 2009). Any alteration of the learning component (i.e., the capacity of association between food stimulus and environment) could be associated with alterations of food reward.

Even if each component seems to activate distinct circuits in the reward system, it is mostly impossible to really separate them during food behaviors analysis. In the literature, there is still controversy about this discrimination (Garbinsky et al., 2014; Jiang et al., 2015; Polk et al., 2017). For example, the Leeds Food Preference Questionnaire permits an analysis of an explicit wanting and an implicit liking in humans. For the wanting component, participants are asking to answer to “which food do you most want to eat now?” between two food pictures (Finlayson et al., 2008, 2011).

#### In physiopathology (obesity)

In 2001, Wang et al. (2001) were the first to show a dysregulation of the dopaminergic reward system in obese humans. Several studies have shown that long-term overeating in both rodents and humans induces a decrease in the dopamine released, in the dopamine turnover (ratio between DOPAC and dopamine), in the dopamine receptors 1 and 2 (DRD1 and DRD2) and an increase in the DAT (Alastair et al., 2015; Décarie-Spain et al., 2016; for more information see reviews Alastair et al., 2015; Décarie-Spain et al., 2016).

In the context of obesity, altered food reward behaviors have been demonstrated in preclinical models. Studies have proven that rats under high-fat diet pressed less on the lever during the operant conditioning test to obtain a food reward compared to lean rats, suggesting an alteration of the wanting component. During the conditioned place preference, these obese rats spent less time in the compartment associated with palatable food than lean mice. Therefore, the learning component seems also to be dysregulated in the context of obesity (Davis et al., 2008; Tracy et al., 2015). Furthermore, several studies demonstrated that obese mice present a decrease in the preference for palatable food compared to lean mice, suggesting an alteration of the liking component in the context of obesity (Vucetic et al., 2011; Carlin et al., 2013).

Epstein et al. (2007) and Stice et al. (2008b) have proved that obese humans show a decrease of the striatal activation in response to food reward consumption compared to lean humans. These altered food reward behaviors are often called food addiction, because of neuronal and behavioral similarities with drug addiction, as well as neuronal adaptations. After the stimulation with repeated palatable stimuli, the individuals need to increase the size of the portion of food ingested to obtain the same pleasure, for the same food reward stimulation, obese individuals will show less striatal activation. However, the scientific community is not yet unanimously agreed on the term food addiction (Kenny, 2011; Wiss et al., 2018; Campana et al., 2019).

One human genetic-related condition—TaqIA A1 allele polymorphism—associated with altered reward process is particularly interesting to study and to understand the underlying biological mechanisms. The TaqIA A1 allele polymorphism affects 30–40% of the population and is associated with 30–40% decrease in the DRD2 abundance in the striatum (Stice et al., 2010). These neuronal changes correlate with addiction and compulsive behavior (Wang et al., 2001; Stice et al., 2008a). One study showed that this genetic susceptibility was present in 67% of overweight or obese subjects included in the study (Chen et al., 2012).

Importantly, even if the homeostatic and non-homeostatic systems are described separately, these systems work in concert to control food intake. For instance, Figlewicz et al. (2003) have demonstrated that dopaminergic neurons in the VTA expresses insulin and leptin receptors and several studies have shown their implications in the food reward. Indeed, mice with a specific leptin receptor long-term RNA mediated knockdown in the VTA present an increase in the palatable food intake (Hommel et al., 2006). Moreover, it has been described that mesencephalic dopamine neurons coexpress CCK (Crawley, 1991; Hokfelt et al., 2002). Therefore, it is now suggested that these systems need to be simultaneously investigated instead of separately.

## Gut microbiota and the control of food intake

**FIGURE 3:**
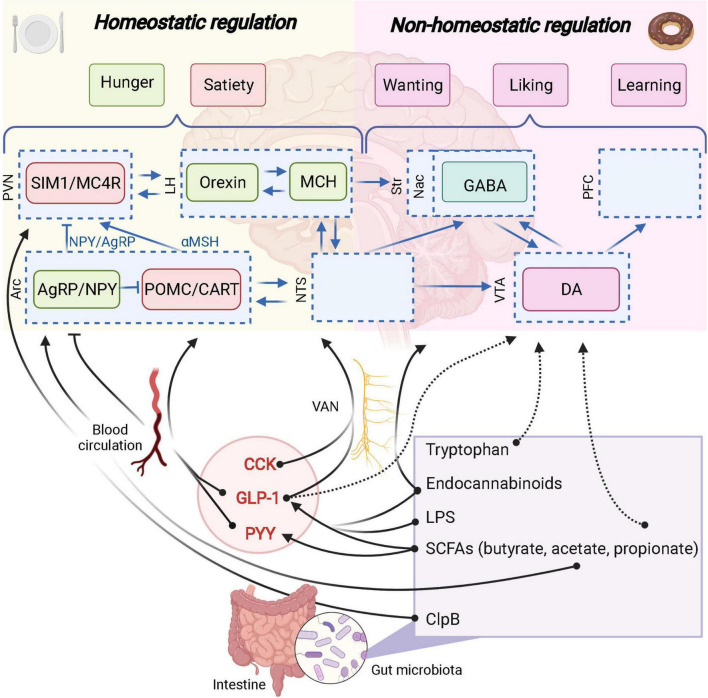
The role of the gut microbiota in homeostatic and non-homeostatic controls of food intake. Potential links are represented in dashed arrows. Demonstrated links are represented in solid arrows. PVN, paraventricular nucleus; SIM1, single–minded family BHLH transcription factor 1; LH, lateral hypothalamus; Str, striatum; Nac, nucleus accumbens; GABA, γ-aminobutyric acid; PFC, prefrontal cortex; Arc, arcuate nucleus; AgRP, agouti-related protein; NPY, neuropeptide Y; POMC, pro-opiomelanocortin; CART, cocaine-amphetamine-related transcript; NTS, nucleus tractus solitarus; VTA, ventral tegmental area; DA, dopamine; VAN, vagal afferent nerves; CCK, cholecystokinin; GLP-1, glucagon-like peptide 1; PYY, peptide YY; SCFAs, short-chain fatty acids; LPS, lipopolysaccharide; MC4R, Melanocortin 4 receptor. Created with BioRender.com.

The gut microbiome includes microorganisms (bacteria, archaea, viruses, yeast, and fungi) that colonize the gut, their genomes and metabolites. The gut microbiota has emerged as a key player into the gut-brain axis through the vagal and humoral pathways to modulate host metabolism and food intake (Figure 3; Delzenne et al., 2011; van de Wouw et al., 2017; Cani et al., 2019). One pioneer study showed in 2010, by fecal material transplantation, the causal role of the gut microbiota into eating behavior alterations (hyperphagia) associated with the obese phenotype of Toll-like receptor 5 (TLR5) knockout mice (Vijay-Kumar et al., 2010). Several tools have been developed to modulate the gut microbiota composition though different approaches: by the administration of microbes (beneficial microbes or probiotic as defined in Table 1) or by dietary intervention with specific substrate for gut microbes (prebiotic as defined in Table 1). Importantly, some of these approaches have been shown to impact host food intake. Moreover, some gut microbiota components and metabolites have also been described to be involved in the homeostatic control of food intake.

**TABLE 1:** Definitions of prebiotic (Gibson et al., 2017) and probiotic (Hill et al., 2014).

Probiotic	Live microorganisms that, when administered in adequate amounts, confer a health benefit on the host
Prebiotic	A substrate that is selectively utilized by host microorganisms conferring a health benefit

Created with BioRender.com.

### Gut microbiota and homeostatic control of food intake

In this part of the review, we will focus mainly on the L cells and the products of these EECs that have also been directly linked with the control of food intake and whose concentrations are under the possible control of the gut microbiota.

#### Enteroendocrine cells and gut microbes: Impact on glucagon-like peptide-1 and peptide YY

To the best of our knowledge, the earliest demonstration describing specific changes in EECs physiology and linked to gut microbes came from experiments using germ-free (GF) animals. In a first study published in 1989, it was shown that the circulating levels of “enteroglucagon” and PYY were much higher in animals raised without microbes compared to conventionally raised animals (Goodlad et al., 1989). Surprisingly, in the proximal small intestine, protein levels of key peptide the GLP-1 and PYY were found to be significantly lower in GF mice (Duca et al., 2012). In the same study, the density of the EECs was lower in the ileum while higher in the colon of GF mice.

Wichmann et al. (2013) aimed at deciphering how the gut microbiota could influence the GLP-1 producing cells and confirmed the increased expression of proglucagon gene, increased GLP-1-positive cell number in the colon and higher circulating GLP-1 in GF mice vs. conventionalized mice. Various dietary polysaccharides (e.g., dietary fibers) are not digested by the host enzymes and part of them are metabolized by gut microbes through anaerobic fermentation. A substantial proportion of these non-digestible compounds will be used as an energy source by specific gut bacteria thereby contributing to the production of different metabolites, such as the short chain fatty acids (SCFAs) (e.g., acetate, butyrate and propionate) (Louis and Flint, 2016). Interestingly, monocolonization of the intestine of GF mice with *Escherichia coli* (which does not produce SCFAs) does not affect proglucagon expression or L cell number, while using a known SCFAs producer such as *Bacteroides thetaiotaomicron* is significantly affecting these parameters. Altogether, these data suggest that SCFAs are involved in the dialogue between gut microbes and EEC cells (Figure 3; Wichmann et al., 2013).

More than 20 years ago, our laboratory discovered in either genetically or diet-induced obesity rodents that treating them with a diet enriched with prebiotics such as inulin or oligofructose (i.e., inulin-type fructans) modulates the gut microbes and improves metabolic parameters together with decreasing food intake (Daubioul et al., 2000; Cani et al., 2004, 2005, 2006; Delzenne et al., 2005). Seeking for the molecular mechanism that explains the lower food intake and improved glucose tolerance, the improved phenotype was associated with higher GLP-1 content and proglucagon mRNA expression in the intestinal segments and higher levels of GLP-1 in the portal vein blood (Cani et al., 2004, 2005, 2006). In the same line, deleting the production of PYY is linked with obesity and hyperphagia in mice (Batterham et al., 2006), whereas prebiotic treatment is associated with increased level of PYY in the circulation (Delzenne et al., 2005).

Later, we discovered that these effects were accompanied by an increased number of EECs L-cells (Cani et al., 2007b; Everard et al., 2011). Of note, these properties were not limited to inulin-type fructans since resistant starches and arabinoxylans also fermentable and modifying the microbiota, decreased food intake, fat mass and body weight gain, together with increased plasma GLP-1 and PYY levels (Zhou et al., 2008; Aziz et al., 2009). Similarly, the microbial fermentation of these other fiber compounds led to the production of SCFAs.

#### Short chain fatty acids

Approximatively 100–150 mmol/kg of SCFAs can be found in the caecum and colon. Acetate, propionate and butyrate represent a ratio of 60/20/20. Besides their local effects in the intestine part of them can impact others organs located at distance of the gut including the brain (Frost et al., 2014; Canfora et al., 2015).

On a mechanistic point of view, there are several effectors linking SCFAs and the secretion of gut peptides from the EEC. Indeed, SCFAs have been shown to bind and activate the free fatty acid receptor 2 and 3 (FFAR2 and FFAR3) also known as specific G-protein-coupled receptors 43 and 41, respectively (GPR-43 and GPR-41) (Brown et al., 2003). Importantly, both GPR-41 and GPR-43 are expressed on EEC L-cells and are directly activated by the SCFAs produced by the gut microbiota and eventually promote the secretion of gut peptides such as GLP-1 and PYY (Figure 3; de Vos et al., 2022). Of note, mice lacking these receptors display altered secretion of GLP-1 and PYY after exposure to SCFAs or specific prebiotics (Psichas et al., 2015; Koh et al., 2016; Brooks et al., 2017).

Various bacterial species are changed after prebiotic supplementation. Ten years ago, we discovered that prebiotics also strongly increased the abundance of *Akkermansia muciniphila* (Everard et al., 2011). Strikingly, this bacterium was almost lacking in obese/diabetic mice, while prebiotics restored its abundance (Everard et al., 2013). *A. muciniphila* can produce propionate and acetate, both acting as agonists for agonists GPR-41, GPR-43 (Derrien et al., 2004). We discovered that the administration of *A. muciniphila* exerts protective effects against diet-induced obesity, diabetes, hepatic steatosis, gut barrier dysfunction and in specific models of increased caloric intake (Everard et al., 2013, 2019; Plovier et al., 2017; Depommier et al., 2020).

In a therapeutic point of view, there is a great therapeutic interest in modulating the production of SCFAs by using the gut microbiota. Indeed, targeting the endogenous production of GLP-1 and PYY is highly attractive since the levels of these peptides are decreased in obese and diabetic individuals (Ranganath et al., 1996; Mannucci et al., 2000; Verdich et al., 2001; Vilsboll et al., 2001; Batterham et al., 2003; le Roux et al., 2006). In overweight adults, the supplementation in inulin-propionate ester (delivering specifically propionate in the colon) not only increased PYY and GLP-1 plasma concentrations, but decreased also significantly energy intake, leading to weight loss (Chambers et al., 2015). Moreover, deleting the production of PYY is linked with obesity and hyperphagia in mice (Batterham et al., 2006).

In addition to the effects of SCFAs in the intestine, SCFAs themselves are also able to enter the circulation through the portal vein (Cummings et al., 1987; Weaver et al., 1988) and eventually reach organ at distance including the brain (Frost et al., 2014; Canfora et al., 2015). It has been shown that gut-derived acetate is able to reach the brain and impact food behavior suppressing appetite (Frost et al., 2014).

#### Endocannabinoids

As discussed here above, prebiotic supplementation was associated with SCFAs production and correlated with the production of GLP-1/PYY. However, other mediators are involved in this crosstalk between gut microbes and host food intake. Interestingly, the administration of *A. muciniphila* can also increases the levels of specific bioactive lipids related to endocannabinoids (Figure 3) (i.e., *N*-oleoylethanolamine (OEA), 2-oleoylglycerol (2-OG), 2-arachidonoylglycerol [2-AG) and 1 and 2 palmitoylglycerol (1-PG/2-PG)] (Everard et al., 2013; Depommier et al., 2021). Some of these lipids, including OEA and 2-OG have been shown to trigger the secretion of GLP-1 by activating the G-coupled receptor 119 (GPR119) (Figure 3; Lan et al., 2009; Lauffer et al., 2009; Hansen et al., 2011; Moss et al., 2015). This suggests that *A. muciniphila* exerts beneficial effects on the metabolic health of its host by modulating the production of GLP-1 through different metabolic products. More recently the role of the protein P9 specifically produced by *A. muciniphila* has also been proposed and shown to promote the secretion of GLP-1 by stimulating the L-cells *via* a different mechanism than GPR43/41 or GPR119-dependent pathways (Cani and Knauf, 2021; Yoon et al., 2021).

Besides the role of *A. muciniphila*, it has been discovered that the human gut microbiota can produce *N*-acyl amides. Those bacterial metabolites are mimicking endocannabinoid-like lipids with some of them activating some endocannabinoid related receptors such as GPR119 (Cohen et al., 2017). Therefore, activating GPR119 present on the L-cells triggers GLP-1 secretion and eventually may control food intake, glucose, and energy metabolism (Cohen et al., 2017; Yang et al., 2018). More recently, by using in GF mice an extensive analysis of the endocannabinoidome (eCBome) which comprises numerous bioactive lipid families biochemically related to endocannabinoids, their receptors, and metabolic enzymes, we discovered that the gut microbiota plays an important role on the regulation of this system and this on numerous organs and targets including the EEC and their products (Manca et al., 2020a).

The eCBome is widely distributed in various tissues and organs including the hypothalamus and different areas of the brain but also in the liver, the gut, and the adipose tissues. Therefore, the eCBome is now viewed as a key target to modulate numerous physiological functions such as the control of food intake but also glucose/lipid metabolism and inflammation among other pathways. Hence, understanding the link between the eCBome and more specifically the role of the gut microbiota on this system constitutes an important target.

#### Lipopolysaccharide

Gut barrier plays a key role in the symbiotic relationship between gut microbes and the host since it allows the uptake of essential nutrients and immune sensing, while being restrictive against pathogenic molecules and bacteria (Regnier et al., 2021). Indeed, alterations of the gut barrier, facilitates lipopolysaccharide (LPS) translocation into the circulation. This increase in plasma lipopolysaccharide (LPS) (defined as metabolic endotoxemia) is involved in the onset of low-grade inflammation characterizing obesity (Cani et al., 2007a). LPS induces the activation of pro-inflammatory pathways mainly through TLR4 in several organs including the brain. Interestingly hypothalamic inflammation has been associated with homeostatic food intake alterations (Thaler et al., 2013).

That said, it is important to precise that even before the onset of inflammation, the administration of LPS has been demonstrated to stimulate the secretion of GLP-1 from EECs through a TLR4-dependent mechanism (Figure 3; Lebrun et al., 2017).

#### Caseinolytic peptidase B

Some bacterial components are also able to enter the blood circulation to reach the brain. It has been described for the caseinolytic peptidase B (ClpB), a chaperone protein from *Escherichia coli*, that mimics anorexigenic hormone α-MSH and binds to its receptor MC4R (Figure 3; Tennoune et al., 2014). This protein has been associated with eating disorders (anorexia nervosa, bulimia, binge-eating disorders) and obesity in human populations (Tennoune et al., 2014).

### Gut microbiota and non-homeostatic control of food intake

As described above, the control of non-homeostatic food intake is mainly driven by dopaminergic neurons at the level of the mesocorticolimbic brain areas. Interestingly, animal models depleted in gut microbiota have demonstrated the key role of gut microbes in the dopaminergic transmission. In one study, GF mice showed increased synthetizing dopamine turnover (ratio DOPAC/dopamine) in the striatum as compared to conventional specific pathogen free (SPF) mice (Diaz Heijtz et al., 2011). Another study showed that GF rats have lower dopamine degradation turnover rate (ratio HVA/dopamine) in the striatum as compared to SPF mice (Crumeyrolle-Arias et al., 2014). These results were further confirmed with another approach of gut microbiota depletion: mice treated with antibiotics (Desbonnet et al., 2015). These pioneering studies opened the field for the investigation of the role of gut microbes in many dopaminergic-related disease including Parkinson’s disease, addictions or hedonic dysregulations associated with obesity (Hamamah et al., 2022).

The impacts of the alterations of the dopaminergic system in absence of gut microbiota were then confirmed on a food intake and more precisely on behavior. Indeed, GF mice show increased hedonic intake (increased preference for lipid solution and increased intake of highly concentrated sucrose solution) compared to control mice (Duca et al., 2012; Swartz et al., 2012). More recently, we showed that lean antibiotic-treated mice eat significantly more high-fat high-sucrose (HFHS) diet during a food preference test compared to lean mice with intact gut microbiota (de Wouters d’Oplinter et al., 2021). Even if these studies identified a role of gut microbes in hedonic feeding, our team were the first to establish the proof-of concept of the causal role of gut microbes in altered hedonic food intake associated with obesity (de Wouters d’Oplinter et al., 2021). After transferring the fecal material from obese mice into lean recipient mice, obese gut microbiota recipient mice behave as their obese donors: they eat less palatable food (HFHS) than lean gut microbiota recipient mice. We also showed that mice transferred from obese gut microbiota donors have similar dopaminergic alterations as observed during obesity, including reduced expressions of DRD1 and DRD2, TH and increased expression of DAT in the striatum as compared to mice receiving gut microbiota from lean mice. These data demonstrate the causal roles of gut microbes in the alteration of dopaminergic mesocorticolimbic system during obesity.

#### Amino acids metabolism

The gut microbiota modulates the intestinal production of neurotransmitters involved in the reward system including serotonin and dopamine from key amino acids precursors such as tryptophan, phenylalanine and tyrosine (Strandwitz, 2018). For instance, it has been shown that gut microbes increase colonic expression of tryptophan hydroxylase 1, the rate limiting enzyme synthetizing serotonin from tryptophan (Reigstad et al., 2015). However, the impacts of intestinal amino acids modulation on the brain and in particular on the food reward system still needs to be address.

Moreover, amino acids themselves can influence the gut microbiota composition and the reward system through the gut-brain axis. Indeed, tryptophan has not only homeostatic anorexigenic properties (shown on the hypothalamus, in hungry conditions), but has also inhibiting effects on palatable food intake. In fact, intra gastric infusion of tryptophan reduced palatable intake in satiated mice and was associated with decreased cFOS expression in the Nac shell and core (Figure 3; Gartner et al., 2018).

Recently, a large-scale clinical study strengthened the key associations between fecal amino acids, the gut microbiota and the reward system in the context of obesity (Dong et al., 2022). After confirming the alterations of the reward system in obesity at the level of the Nac, the authors characterized the obese gut microbiota by a higher *Prevotella/Bacteroides* ratio. They further linked this ratio with nucleus accumbens alterations and fecal tryptophan, with significant interactions. In this model, subjects with higher body-mass index, would have higher *Prevotella/Bacteroides* ratio and decreased fecal tryptophan. Whether if the link between reduced tryptophan and alterations of the reward system is mediated by modulations of neurotransmitters remains to be demonstrated. Another human study showed a negative association between bacterial gene functionality coding for the enzyme synthetizing phenylalanine, the precursor of dopamine, and the ventral striatal functional MRI response during reward anticipation (Aarts et al., 2017). These results suggest a potential role of gut microbes in neurotransmitters production in the intestine and their impacts on the brain. However, so far, these were only correlations, the causal role of neurotransmitters modulated by the gut microbiota on the reward system remains to be demonstrated.

#### Vagus nerve

Reward-signaling pathways from the gut to the brain have been highlighted thanks to the development of optogenetic technologies and vagotomy protocols. In 2018, Han et al. (2018b) demonstrated that one key mechanism by which the gut communicates with the brain and influence the food reward system is the vagus nerve, and uses glutamate as neurotransmitter. So far, it is not clear if the gut microbiota uses this pathway to influence the food reward system. However, several evidence have been raised in favor of that hypothesis, since vagal afferent nerves expressed receptors for gut-derived compounds such as SCFAs or gut peptides (GLP-1) influencing homeostatic and hedonic food intakes (Figure 3; Egerod et al., 2018; Cook et al., 2021).

#### Glucagon-like peptide-1

Acetate, butyrate and propionate have been broadly described as beneficial gut-derived metabolites in the context of obesity for their impact on food intake through the release of satiation gut peptides GLP-1 and PYY (de Vos et al., 2022). Importantly, homeostatic and non-homeostatic systems can interact and GLP-1 receptors (GLP1R) are also expressed in reward-related areas including the VTA and the Nac (Alhadeff et al., 2012; Decarie-Spain and Kanoski, 2021). Moreover, micro-injections of exendin-4, a GLP-1 analog, in the Nac and in the VTA reduces sucrose and fat intakes, whereas the antagonist of GLP1R, exendin-9 has opposite effects (Alhadeff et al., 2012). The same pharmacological approach demonstrated that GLP1R activation was able to reverse the conditioned place preference-induced behavior and operant conditioning for sucrose reward (Dickson et al., 2012), potentially acting through an inhibition of dopaminergic firing and signaling in the Nac (Wang et al., 2015; Konanur et al., 2020). Therefore, one may suggest that modulation of GLP-1 by gut microbes could also impact the reward system (Figure 3). Of note, GLP-1 is also produced locally in the brain, from preproglucagon neurons (Trapp and Brierley, 2022). The implication of gut-derived GLP-1 from brain-derived GLP-1 on the reward system is not yet clearly established and the roles of gut microbes in the regulation of brain-derived GLP-1 remain to be investigated.

#### Short chain fatty acids

In the context of the reward system, effects of SCFAs can also be observed independently of GLP1 and PYY modulations. Indeed, a small clinical study involving healthy men showed that the administration of inulin-propionate ester, increasing selectively colonic propionate, was associated with reduces anticipatory reward responses in the human striatum to high-energy foods (Byrne et al., 2016). These results were not linked with changes in plasma PYY or GLP-1 suggesting an effect of propionate on the food reward system independent of GLP-1 and PYY (Figure 3). In another context of reward alterations, i.e., the chronic unpredictable mild stress (CUMS), the supplementation in propionate intra-rectally, restore the sucrose preference, together with the concentration of dopamine in the prefrontal cortex of rats (Li et al., 2018). Conversely, another study investigating CUMS showed an increase in sucrose preference after inducing stress and a decrease in sucrose preference upon SCFAs administration (van de Wouw et al., 2018). It is worth noting that in this study, SCFAs administration did not change SCFAs levels in the feces.

#### Endocannabinoids

Endocannabinoids have been described as important neurotransmitters involved in the food reward system mainly through the activation of cannabinoid receptor 1 (CB1) and 2 (CB2) in the brain and in the gut, and by mediating fat preference (DiPatrizio et al., 2013; Lau et al., 2017). The vagus nerve implication in the effects of endocannabinoids on the reward-related processes has been demonstrated in this context (Figure 3). Indeed, a recent study showed that blocking peripheral CB1 receptors decreased dopaminergic neurons activation and palatable food intake though the vagus nerve (Berland et al., 2022). The vagal pathway is also token by other bioactive lipids related to the endocannabinoid such as OEA. This a gut-derived endocannabinoid was identified as an intestinal lipid sensor, to increase striatal dopamine release, and decrease high-fat intake in favor of low-fat solution intake (Tellez et al., 2013; Hankir et al., 2017).

More studies need to investigate respective effects of other endocannabinoids on hedonic food intake. One study in binge-eating subjects showed that plasma levels of anandamide was significantly elevated after eating palatable food without changes in 2-AG levels (Monteleone et al., 2017); while another one in rodents showed that 2-AG was elevated after binge-eating episode (Berland et al., 2022).

Interestingly since gut microbes are able to modulate endocannabinoids and related lipids they could represent potential mediators between gut microbes and food reward. Indeed, GF mice exhibited altered brain and intestinal endocannabinoidome (i.e., bioactive lipids and their receptors), while these effects are reversed after fecal material transplantation (Manca et al., 2020a,b). It is clear that endocannabinoids signal through the vagus nerve to influence the reward system as well as that gut microbes are able to modulate endocannabinoids and related lipids both in the intestine and the gut (Manca et al., 2020a). However, the direct role of gut microbes through endocannabinoid lipids on the food reward system need to be demonstrated. Taken together, these results suggest that endocannabinoids represent potential mediators between the gut microbiota and the food reward system (Figure 3).

#### Inflammation

Among the mechanisms underlying food reward alterations, inflammation has emerged as a promising pathway (Sun et al., 2017). Activation of pro-inflammatory pathways in reward-related areas (Nac) has been described after high-fat feeding, together with impaired motivation for sucrose rewards during an operant conditioning task (Décarie-Spain et al., 2018). Interestingly, silencing an important pathway of LPS inducing inflammation, that is nuclear factor-κB (NFκB) pathway, decreases the compulsive sucrose seeking (Décarie-Spain et al., 2018). Alterations in the gut microbiota composition has been identified to mediate inflammation through the systemic circulation and the brain, notably through the disruption of the gut and blood-brain barrier integrity (Cani et al., 2008; Everard et al., 2013; Braniste et al., 2014; Erny et al., 2015). More precisely, in the Nac, the inflammation induced by a high-fat diet is reversed after antibiotic treatment and transferable by fecal transplantation (Soto et al., 2018). The role of the gut microbiota in inflammation-mediated behavioral alterations of the food reward system still needs to be answered.

In a mechanistic point of view, it has been shown that modulation of the gut microbiota by the administration of SCFAs restores microglia (immune cells of the brain) malformation and immaturity of the cortex of GF mice (Erny et al., 2015). In addition, the gut microbes play key roles in the maintain of the blood brain barrier which acts as a crucial gatekeeper to control the passage of molecules between the circulatory system and the brain, including pro inflammatory molecules. Therefore, the maintain of an appropriate BBB by the gut microbiota represents an important mechanism of protection during inflammation (Braniste et al., 2014).

## Conclusion and therapeutic perspectives

In the treatment of eating disorders such as binge eating, strongly associated with obesity, only few pharmacological approaches appear to be effective (Aguera et al., 2021). Considering the gut microbiota interactions with the food reward system previously described, the modulation of the gut microbiota seems promising to treat eating disorders. However, so far, only few studies have investigated this approach.

Some interesting associations were found between the use of *Bifidobacterium infantis* and dopamine modulation in reward related area of the brain, however, its impacts on food reward remains to be demonstrated (Desbonnet et al., 2008). One study found an inverse correlation between the abundance of *Akkermansia muciniphila* and the food addiction score in women (Dong et al., 2020). *Akkermansia muciniphila* acts on multiple pathways involved in the reward system including inflammation, the production of SCFA, the endocannabinoid tone, making it a good candidate in the regulation of food reward alterations. Some interesting associations were also found about *Bifidobacterium infantis.* Its supplementation revealed anti-inflammatory properties in rodents, coupled with increased plasma concentrations of tryptophan and decrease DOPAC in the cortical area of the brain (Desbonnet et al., 2015). Another potential beneficial bacterium in this context is *Bifidobacterium pseudocatenulatum.* Indeed, this bacterium influences hedonic food behavior by reversing HFD-induced reduction in sucrose preference (Agusti et al., 2018). Other eating disorders related to the reward system such as binge eating are also associated with obesity. The same research group also investigated the administration of *Bacteroides uniformis* in a binge eating model of mice. Its supplementation decreases the total caloric intake of the binge episode, but with no difference in hedonic intake (sucrose solution). Besides, *Bacteroides uniformis* was associated with an increased expression of dopamine receptor 1 in the prefrontal cortex (Agusti et al., 2021). However, the effects of gut microbes on other components of the food reward as well as the effect of other beneficial microbes and the mechanisms related to these effects still need to be investigated.

The prebiotic approach modifies the gut microbiota composition with beneficial effects on the host, notably through the production of SCFAs. If their effects on homeostatic food intake are consistent, it is not the case in hedonic mechanisms. On the one hand, the administration of fructo-oligosaccharides to diet-induced obese mice decreased their motivation for sucrose pellets as compared to diet-induced obese mice, after an overnight fasting, suggesting a worsening of their wanting component of the reward (Delbes et al., 2018). These behavioral results were not associated with changes in dopaminergic markers expressions in the nucleus accumbens. On the other hand, another study using prebiotics, has shown that inulin (a long chain fructan) increases sucrose preference and ameliorates gut microbiota composition alterations induced by a high-fat diet (Bernard et al., 2019). Finally, an oligosaccharide from the human milk namely the 2-fucosyllactose, has been shown to potentiate the motivation for food reward through the vagus nerve (Vazquez et al., 2016).

In conclusion, we demonstrated the causal roles of gut microbes in food reward alterations associated with obesity and combined with these studies altogether this support the interest of using gut microbiota targeted approaches to modulate food reward behavior that is altered during obesity the related mechanism remain to be addressed.

## Author contributions

AE and AdW designed and conceived the outline of the review. All authors have equally contributed to the writing.

## References

[B1] Aarts E. Ederveen T. H. A. Naaijen J. Zwiers M. P. Boekhorst J. Timmerman H. M. (2017). Gut microbiome in ADHD and its relation to neural reward anticipation. *PLoS One* 12:e0183509. 10.1371/journal.pone.0183509 28863139 PMC5581161

[B2] Abbott C. R. Monteiro M. Small C. J. Sajedi A. Smith K. L. Parkinson J. R. (2005). The inhibitory effects of peripheral administration of peptide YY(3-36) and glucagon-like peptide-1 on food intake are attenuated by ablation of the vagal-brainstem-hypothalamic pathway. *Brain Res.* 1044 127–131. 10.1016/j.brainres.2005.03.011 15862798

[B3] Acuna-Goycolea C. van den Pol A. N. (2005). Peptide YY(3-36) inhibits both anorexigenic proopiomelanocortin and orexigenic neuropeptide Y neurons: implications for hypothalamic regulation of energy homeostasis. *J. Neurosci.* 25 10510–10519. 10.1523/JNEUROSCI.2552-05.2005 16280589 PMC6725817

[B4] Aguera Z. Lozano-Madrid M. Mallorqui-Bague N. Jimenez-Murcia S. Menchon J. M. Fernandez-Aranda F. (2021). A review of binge eating disorder and obesity. *Neuropsychiatr* 35 57–67. 10.1007/s40211-020-00346-w 32346850

[B5] Agusti A. Campillo I. Balzano T. Benitez-Paez A. Lopez-Almela I. Romani-Perez M. (2021). *Bacteroides* uniformis CECT 7771 Modulates the Brain Reward Response to Reduce Binge Eating and Anxiety-Like Behavior in Rat. *Mol. Neurobiol.* 58 4959–4979. 10.1007/s12035-021-02462-2 34228269 PMC8497301

[B6] Agusti A. Moya-Perez A. Campillo I. Montserrat-De La Paz S. Cerrudo V. Perez-Villalba A. (2018). Bifidobacterium pseudocatenulatum CECT 7765 Ameliorates Neuroendocrine Alterations Associated with an Exaggerated Stress Response and Anhedonia in Obese Mice. *Mol. Neurobiol.* 55 5337–5352. 10.1007/s12035-017-0768-z 28921462

[B7] Alastair J. Tulloch S. M. Regina Vaicekonyte, Nicole M. Avena. (2015). Food and the brain : how the brain responds to nutrients. *Gastroenterology* 148 1205–1218. 10.1053/j.gastro.2014.12.058 25644095

[B8] Albuquerque D. Nobrega C. Manco L. Padez C. (2017). The contribution of genetics and environment to obesity. *Br. Med. Bull.* 123 159–173. 10.1093/bmb/ldx022 28910990

[B9] Alhadeff A. L. Rupprecht L. E. Hayes M. R. (2012). GLP-1 neurons in the nucleus of the solitary tract project directly to the ventral tegmental area and nucleus accumbens to control for food intake. *Endocrinology* 153 647–658. 10.1210/en.2011-1443 22128031 PMC3275387

[B10] Arias-Carrion O. Stamelou M. Murillo-Rodriguez E. Menendez-Gonzalez M. Poppel E. (2010). Dopaminergic reward system: a short integrative review. *Int. Arch. Med.* 3:24. 10.1186/1755-7682-3-24 20925949 PMC2958859

[B11] Asakawa A. Inui A. Kaga T. Yuzuriha H. Nagata T. Ueno N. (2001). Ghrelin is an appetite-stimulatory signal from stomach with structural resemblance to motilin. *Gastroenterology* 120 337–345. 10.1053/gast.2001.22158 11159873

[B12] Aziz A. A. Kenney L. S. Goulet B. Abdel-Aal E. (2009). Dietary starch type affects body weight and glycemic control in freely fed but not energy-restricted obese rats. *J. Nutr.* 139 1881–1889. 10.3945/jn.109.110650 19692526

[B13] Batterham R. L. Cohen M. A. Ellis S. M. Le Roux C. W. Withers D. J. Frost G. S. (2003). Inhibition of food intake in obese subjects by peptide Yy3-36. *N. Engl. J. Med.* 349 941–948. 10.1056/NEJMoa030204 12954742

[B14] Batterham R. L. Heffron H. Kapoor S. Chivers J. E. Chandarana K. Herzog H. (2006). Critical role for peptide Yy in protein-mediated satiation and body-weight regulation. *Cell Metab.* 4 223–233. 10.1016/j.cmet.2006.08.001 16950139

[B15] Berland C. Castel J. Terrasi R. Montalban E. Foppen E. Martin C. (2022). Identification of an endocannabinoid gut-brain vagal mechanism controlling food reward and energy homeostasis. *Mol. Psychiatry* 27 2340–2354. 10.1038/s41380-021-01428-z 35075269

[B16] Bernard A. Ancel D. Neyrinck A. M. Dastugue A. Bindels L. B. Delzenne N. M. (2019). A preventive prebiotic supplementation improves the sweet taste perception in diet-induced obese mice. *Nutrients* 11:549. 10.3390/nu11030549 30841548 PMC6471995

[B17] Berthoud H. R. Blackshaw L. A. Brookes S. J. H. Grundy D. (2004). Neuroanatomy of extrinsic afferents supplying the gastrointestinal tract. *Neurogastroenterol. Motil.* 16 28–33. 10.1111/j.1743-3150.2004.00471.x 15066001

[B18] Berthoud H. R. Munzberg H. Morrison C. D. (2017). Blaming the brain for obesity: integration of hedonic and homeostatic mechanisms. *Gastroenterology* 152 1728–1738. 10.1053/j.gastro.2016.12.050 28192106 PMC5406238

[B19] Bottcher G. Ekblad E. Ekman R. Hakanson R. Sundler F. (1993). Peptide Yy: a neuropeptide in the gut. Immunocytochemical and immunochemical evidence. *Neuroscience* 55 281–290. 10.1016/0306-4522(93)90472-r 8350990

[B20] Braniste V. Al-Asmakh M. Kowal C. Anuar F. Abbaspour A. Toth M. (2014). The gut microbiota influences blood-brain barrier permeability in mice. *Sci. Transl. Med.* 6:263ra158. 10.1126/scitranslmed.3009759 25411471 PMC4396848

[B21] Brooks L. Viardot A. Tsakmaki A. Stolarczyk E. Howard J. K. Cani P. D. (2017). Fermentable carbohydrate stimulates Ffar2-dependent colonic Pyy cell expansion to increase satiety. *Mol. Metab.* 6 48–60. 10.1016/j.molmet.2016.10.011 28123937 PMC5220466

[B22] Brown A. J. Goldsworthy S. M. Barnes A. A. Eilert M. M. Tcheang L. Daniels D. (2003). The Orphan G protein-coupled receptors Gpr41 and Gpr43 are activated by propionate and other short chain carboxylic acids. *J. Biol. Chem.* 278 11312–11319. 10.1074/jbc.M211609200 12496283

[B23] Bruning J. C. Gautam D. Burks D. J. Gillette J. Schubert M. Orban P. C. (2000). Role of brain insulin receptor in control of body weight and reproduction. *Science* 289 2122–2125. 10.1126/science.289.5487.2122 11000114

[B24] Buccafusco J. J. (2009). *Methods of Behavior Analysis in Neuroscience.* Oxfordshire: Taylor & Francis. 10.1201/9780367802622 21204335

[B25] Byrne C. S. Chambers E. S. Alhabeeb H. Chhina N. Morrison D. J. Preston T. (2016). Increased colonic propionate reduces anticipatory reward responses in the human striatum to high-energy foods. *Am. J. Clin. Nutr.* 104 5–14. 10.3945/ajcn.115.126706 27169834 PMC4919527

[B26] Campana B. Brasiel P. G. De Aguiar A. S. Dutra S. C. P. L. (2019). Obesity and food addiction: similarities to drug addiction. *Obes. Med.* 16:100136. 10.1016/j.obmed.2019.100136

[B27] Canfora E. E. Jocken J. W. Blaak E. E. (2015). Short-chain fatty acids in control of body weight and insulin sensitivity. *Nat. Rev. Endocrinol.* 11 577–591. 10.1038/nrendo.2015.128 26260141

[B28] Cani P. D. Amar J. Iglesias M. A. Poggi M. Knauf C. Bastelica D. (2007a). Metabolic endotoxemia initiates obesity and insulin resistance. *Diabetes* 56 1761–1772. 10.2337/db06-1491 17456850

[B29] Cani P. D. Hoste S. Guiot Y. Delzenne N. M. (2007b). Dietary non-digestible carbohydrates promote L-cell differentiation in the proximal colon of rats. *Br. J. Nutr.* 98 32–37. 10.1017/S0007114507691648 17367575

[B30] Cani P. D. Knauf C. (2021). A newly identified protein from *Akkermansia muciniphila* stimulates Glp-1 secretion. *Cell Metab.* 33 1073–1075. 10.1016/j.cmet.2021.05.004 34077715

[B31] Cani P. D. Bibiloni R. Knauf C. Waget A. Neyrinck A. M. Delzenne N. M. (2008). Changes in gut microbiota control metabolic endotoxemia-induced inflammation in high-fat diet-induced obesity and diabetes in mice. *Diabetes* 57 1470–1481. 10.2337/db07-1403 18305141

[B32] Cani P. D. Dewever C. Delzenne N. M. (2004). Inulin-type fructans modulate gastrointestinal peptides involved in appetite regulation (glucagon-like peptide-1 and ghrelin) in rats. *Br. J. Nutr.* 92 521–526. 10.1079/BJN20041225 15469657

[B33] Cani P. D. Knauf C. Iglesias M. A. Drucker D. J. Delzenne N. M. Burcelin R. (2006). Improvement of glucose tolerance and hepatic insulin sensitivity by oligofructose requires a functional glucagon-like peptide 1 receptor. *Diabetes* 55 1484–1490. 10.2337/db05-1360 16644709

[B34] Cani P. D. Neyrinck A. M. Maton N. Delzenne N. M. (2005). Oligofructose promotes satiety in rats fed a high-fat diet: involvement of glucagon-like Peptide-1. *Obes. Res.* 13 1000–1007. 10.1038/oby.2005.117 15976142

[B35] Cani P. D. Van Hul M. Lefort C. Depommier C. Rastelli M. Everard A. (2019). Microbial regulation of organismal energy homeostasis. *Nat. Metab.* 1 34–46. 10.1038/s42255-018-0017-4 32694818

[B36] Carlin J. Hill-Smith T. E. Lucki I. Reyes T. M. (2013). Reversal of dopamine system dysfunction in response to high-fat diet. *Obesity* 21 2513–2521. 10.1002/oby.20374 23512420 PMC3700634

[B37] Chambers E. S. Viardot A. Psichas A. Morrison D. J. Murphy K. G. Zac-Varghese S. E. (2015). Effects of targeted delivery of propionate to the human colon on appetite regulation, body weight maintenance and adiposity in overweight adults. *Gut* 64 1744–1754. 10.1136/gutjnl-2014-307913 25500202 PMC4680171

[B38] Chen A. L. Blum K. Chen T. J. Giordano J. Downs B. W. Han D. (2012). Correlation of the Taq1 dopamine D2 receptor gene and percent body fat in obese and screened control subjects: a preliminary report. *Food Funct.* 3 40–48. 10.1039/c1fo10089k 22051885

[B39] Chen S. R. Chen H. Zhou J. J. Pradhan G. Sun Y. Pan H. L. (2017). Ghrelin receptors mediate ghrelin-induced excitation of agouti-related protein/neuropeptide Y but not pro-opiomelanocortin neurons. *J. Neurochem.* 142 512–520. 10.1111/jnc.14080 28547758

[B40] Clark R. E. (2018). *Behavioral Neuroscience of Learning and Memory.* New York, NY: Springer 10.1007/978-3-319-78757-2

[B41] Clement K. Vaisse C. Lahlou N. Cabrol S. Pelloux V. Cassuto D. (1998). A mutation in the human leptin receptor gene causes obesity and pituitary dysfunction. *Nature* 392 398–401. 10.1038/32911 9537324

[B42] Cohen L. J. Esterhazy D. Kim S. H. Lemetre C. Aguilar R. R. Gordon E. A. (2017). Commensal bacteria make Gpcr ligands that mimic human signalling molecules. *Nature* 549 48–53. 10.1038/nature23874 28854168 PMC5777231

[B43] Cone R. D. (2005). Anatomy and regulation of the central melanocortin system. *Nat. Neurosci.* 8 571–578. 10.1038/nn1455 15856065

[B44] Considine R. V. Sinha M. K. Heiman M. L. Kriauciunas A. Stephens T. W. Nyce M. R. (1996). Serum immunoreactive-leptin concentrations in normal-weight and obese humans. *N. Engl. J. Med.* 334 292–295. 10.1056/NEJM199602013340503 8532024

[B45] Cook T. M. Gavini C. K. Jesse J. Aubert G. Gornick E. Bonomo R. (2021). Vagal neuron expression of the microbiota-derived metabolite receptor, free fatty acid receptor (Ffar3), is necessary for normal feeding behavior. *Mol. Metab.* 54:101350. 10.1016/j.molmet.2021.101350 34626852 PMC8567301

[B46] Cowley M. A. Smart J. L. Rubinstein M. Cerdan M. G. Diano S. Horvath T. L. (2001). Leptin activates anorexigenic Pomc neurons through a neural network in the arcuate nucleus. *Nature* 411 480–484. 10.1038/35078085 11373681

[B47] Crawley J. N. (1991). Cholecystokinin-dopamine interactions. *Trends Pharmacol. Sci.* 12 232–236. 10.1016/0165-6147(91)90558-A 2048219

[B48] Crumeyrolle-Arias M. Jaglin M. Bruneau A. Vancassel S. Cardona A. Dauge V. (2014). Absence of the gut microbiota enhances anxiety-like behavior and neuroendocrine response to acute stress in rats. *Psychoneuroendocrinology* 42 207–217. 10.1016/j.psyneuen.2014.01.014 24636517

[B49] Cryan J. F. O’riordan K. J. Cowan C. S. M. Sandhu K. V. Bastiaanssen T. F. S. Boehme M. (2019). The Microbiota-Gut-Brain Axis. *Physiol. Rev.* 99 1877–2013. 10.1152/physrev.00018.2018 31460832

[B50] Cummings D. E. Overduin J. (2007). Gastrointestinal regulation of food intake. *J. Clin. Invest.* 117 13–23. 10.1172/JCI30227 17200702 PMC1716217

[B51] Cummings J. H. Pomare E. W. Branch W. J. Naylor C. P. Macfarlane G. T. (1987). Short chain fatty acids in human large intestine, portal, hepatic and venous blood. *Gut* 28 1221–1227. 10.1136/gut.28.10.1221 3678950 PMC1433442

[B52] Daubioul C. A. Taper H. S. De Wispelaere L. D. Delzenne N. M. (2000). Dietary oligofructose lessens hepatic steatosis, but does not prevent hypertriglyceridemia in obese zucker rats. *J. Nutr.* 130 1314–1319. 10.1093/jn/130.5.1314 10801936

[B53] Davis J. F. Tracy A. L. Schurdak J. D. Tschop M. H. Lipton J. W. Clegg D. J. (2008). Exposure to elevated levels of dietary fat attenuates psychostimulant reward and mesolimbic dopamine turnover in the rat. *Behav. Neurosci.* 122 1257–1263. 10.1037/a0013111 19045945 PMC2597276

[B54] de Araujo I. E. Schatzker M. Small D. M. (2020). Rethinking Food Reward. *Annu. Rev. Psychol.* 71 139–164. 10.1146/annurev-psych-122216-011643 31561741

[B55] De Souza C. T. Araujo E. P. Bordin S. Ashimine R. Zollner R. L. Boschero A. C. (2005). Consumption of a fat-rich diet activates a proinflammatory response and induces insulin resistance in the hypothalamus. *Endocrinology* 146 4192–4199. 10.1210/en.2004-1520 16002529

[B56] de Vos W. M. Tilg H. Van Hul M. Cani P. D. (2022). Gut microbiome and health: mechanistic insights. *Gut* 71 1020–1032. 10.1136/gutjnl-2021-326789 35105664 PMC8995832

[B57] de Wouters d’Oplinter A. Rastelli M. Van Hul M. Delzenne N. M. Cani P. D. Everard A. (2021). Gut microbes participate in food preference alterations during obesity. *Gut Microbes* 13:1959242. 10.1080/19490976.2021.1959242 34424831 PMC8386729

[B58] Decarie-Spain L. Kanoski S. E. (2021). Ghrelin and Glucagon-Like Peptide-1: a Gut-Brain Axis Battle for Food Reward. *Nutrients* 13:977. 10.3390/nu13030977 33803053 PMC8002922

[B59] Décarie-Spain L. Hryhorczuk C. Fulton S. (2016). Dopamine signalling adaptations by prolonged high-fat feeding. *Curr. Opin. Behav. Sci.* 9 136–143. 10.1016/j.cobeha.2016.03.010

[B60] Décarie-Spain L. Sharma S. Hryhorczuk C. Issa-Garcia V. Barker P. A. Arbour N. (2018). Nucleus accumbens inflammation mediates anxiodepressive behavior and compulsive sucrose seeking elicited by saturated dietary fat. *Mol. Metab.* 10 1–13. 10.1016/j.molmet.2018.01.018 29454579 PMC5985233

[B61] Delbes A. S. Castel J. Denis R. G. P. Morel C. Quinones M. Everard A. (2018). Prebiotics supplementation impact on the reinforcing and motivational aspect of feeding. *Front. Endocrinol.* 9:273. 10.3389/fendo.2018.00273 29896158 PMC5987188

[B62] Delzenne N. M. Cani P. D. Daubioul C. Neyrinck A. M. (2005). Impact of inulin and oligofructose on gastrointestinal peptides. *Br. J. Nutr.* 93 S157–S161. 10.1079/BJN20041342 15877889

[B63] Delzenne N. M. Neyrinck A. M. Bäckhed F. Cani P. D. (2011). Targeting gut microbiota in obesity: effects of prebiotics and probiotics. *Nat. Rev. Endocrinol.* 7 639–646. 10.1038/nrendo.2011.126 21826100

[B64] Depommier C. Everard A. Druart C. Maiter D. Thissen J. P. Loumaye A. (2021). Serum metabolite profiling yields insights into health promoting effect of *A. muciniphila* in human volunteers with a metabolic syndrome. *Gut Microbes* 13:1994270. 10.1080/19490976.2021.1994270 34812127 PMC8632301

[B65] Depommier C. Van Hul M. Everard A. Delzenne N. M. De Vos W. M. Cani P. D. (2020). Pasteurized *Akkermansia muciniphila* increases whole-body energy expenditure and fecal energy excretion in diet-induced obese mice. *Gut Microbes* 11 1231–1245. 10.1080/19490976.2020.1737307 32167023 PMC7524283

[B66] Derrien M. Vaughan E. E. Plugge C. M. De Vos W. M. (2004). *Akkermansia muciniphila* gen. nov., sp. nov., a human intestinal mucin-degrading bacterium. *Int. J. Syst. Evol. Microbiol.* 54 1469–1476. 10.1099/ijs.0.02873-0 15388697

[B67] Desbonnet L. Clarke G. Traplin A. O’sullivan O. Crispie F. Moloney R. D. (2015). Gut microbiota depletion from early adolescence in mice: implications for brain and behaviour. *Brain Behav. Immun.* 48 165–173. 10.1016/j.bbi.2015.04.004 25866195

[B68] Desbonnet L. Garrett L. Clarke G. Bienenstock J. Dinan T. G. (2008). The probiotic *Bifidobacteria infantis*: an assessment of potential antidepressant properties in the rat. *J. Psychiatr. Res.* 43 164–174. 10.1016/j.jpsychires.2008.03.009 18456279

[B69] Di Marzo V. Ligresti A. Cristino L. (2009). The endocannabinoid system as a link between homoeostatic and hedonic pathways involved in energy balance regulation. *Int. J. Obes.* 33 S18–S24. 10.1038/ijo.2009.67 19528974

[B70] Diaz Heijtz R. Wang S. Anuar F. Qian Y. Bjorkholm B. Samuelsson A. (2011). Normal gut microbiota modulates brain development and behavior. *Proc. Natl. Acad. Sci. U.S.A.* 108 3047–3052. 10.1073/pnas.1010529108 21282636 PMC3041077

[B71] Dickson S. L. Shirazi R. H. Hansson C. Bergquist F. Nissbrandt H. Skibicka K. P. (2012). The glucagon-like peptide 1 (Glp-1) analogue, exendin-4, decreases the rewarding value of food: a new role for mesolimbic Glp-1 receptors. *J. Neurosci.* 32 4812–4820. 10.1523/JNEUROSCI.6326-11.2012 22492036 PMC6620919

[B72] DiPatrizio N. V. Joslin A. Jung K. M. Piomelli D. (2013). Endocannabinoid signaling in the gut mediates preference for dietary unsaturated fats. *FASEB J.* 27 2513–2520. 10.1096/fj.13-227587 23463697 PMC3659363

[B73] Dodd G. T. Tiganis T. (2017). Insulin action in the brain: roles in energy and glucose homeostasis. *J. Neuroendocrinol.* 29:e12513. 10.1111/jne.12513 28758251

[B74] Dong T. S. Guan M. Mayer E. A. Stains J. Liu C. Vora P. (2022). Obesity is associated with a distinct brain-gut microbiome signature that connects *Prevotella* and *Bacteroides* to the brain’s reward center. *Gut Microbes* 14:2051999. 10.1080/19490976.2022.2051999 35311453 PMC8942409

[B75] Dong T. S. Mayer E. A. Osadchiy V. Chang C. Katzka W. Lagishetty V. (2020). A distinct brain-gut-microbiome profile exists for females with obesity and food addiction. *Obesity* 28 1477–1486. 10.1002/oby.22870 32935533 PMC7494955

[B76] Duca F. A. Swartz T. D. Sakar Y. Covasa M. (2012). Increased oral detection, but decreased intestinal signaling for fats in mice lacking gut microbiota. *PLoS One* 7:e39748. 10.1371/journal.pone.0039748 22768116 PMC3387243

[B77] Egerod K. L. Engelstoft M. S. Grunddal K. V. Nohr M. K. Secher A. Sakata I. (2012). A major lineage of enteroendocrine cells coexpress Cck, secretin, Gip, Glp-1, Pyy, and neurotensin but not somatostatin. *Endocrinology* 153 5782–5795. 10.1210/en.2012-1595 23064014 PMC7958714

[B78] Egerod K. L. Petersen N. Timshel P. N. Rekling J. C. Wang Y. Liu Q. (2018). Profiling of G protein-coupled receptors in vagal afferents reveals novel gut-to-brain sensing mechanisms. *Mol. Metab.* 12 62–75. 10.1016/j.molmet.2018.03.016 29673577 PMC6001940

[B79] Epstein L. H. Temple J. L. Neaderhiser B. J. Salis R. J. Erbe R. W. Leddy J. J. (2007). Food reinforcement, the dopamine D2 receptor genotype, and energy intake in obese and nonobese humans. *Behav. Neurosci.* 121 877–886. 10.1037/0735-7044.121.5.877 17907820 PMC2213752

[B80] Erny D. Hrabe De Angelis A. L. Jaitin D. Wieghofer P. Staszewski O. David E. (2015). Host microbiota constantly control maturation and function of microglia in the Cns. *Nat. Neurosci.* 18 965–977. 10.1038/nn.4030 26030851 PMC5528863

[B81] Everard A. Belzer C. Geurts L. Ouwerkerk J. P. Druart C. Bindels L. B. (2013). Cross-talk between *Akkermansia muciniphila* and intestinal epithelium controls diet-induced obesity. *Proc. Natl. Acad. Sci. U.S.A.* 110 9066–9071. 10.1073/pnas.1219451110 23671105 PMC3670398

[B82] Everard A. Lazarevic V. Derrien M. Girard M. Muccioli G. M. Neyrinck A. M. (2011). Responses of gut microbiota and glucose and lipid metabolism to prebiotics in genetic obese and diet-induced leptin-resistant mice. *Diabetes* 60 2775–2786. 10.2337/db11-0227 21933985 PMC3198091

[B83] Everard A. Plovier H. Rastelli M. Van Hul M. De Wouters D’oplinter A. Geurts L. (2019). Intestinal epithelial N-acylphosphatidylethanolamine phospholipase D links dietary fat to metabolic adaptations in obesity and steatosis. *Nat. Commun.* 10:457. 10.1038/s41467-018-08051-7 30692526 PMC6349942

[B84] Farooqi I. S. (2022). Monogenic Obesity Syndromes Provide Insights Into the Hypothalamic Regulation of Appetite and Associated Behaviors. *Biol. Psychiatry* 91 856–859. 10.1016/j.biopsych.2022.01.018 35369984

[B85] Figlewicz D. P. Evans S. B. Murphy J. Hoen M. Baskin D. G. (2003). Expression of receptors for insulin and leptin in the ventral tegmental area/substantia nigra (Vta/Sn) of the rat. *Brain Res.* 964 107–115. 10.1016/S0006-8993(02)04087-8 12573518

[B86] Finlayson G. Arlotti A. Dalton M. King N. Blundell J. E. (2011). Implicit wanting and explicit liking are markers for trait binge eating. A susceptible phenotype for overeating. *Appetite* 57 722–728. 10.1016/j.appet.2011.08.012 21896296

[B87] Finlayson G. King N. Blundell J. (2008). The role of implicit wanting in relation to explicit liking and wanting for food: implications for appetite control. *Appetite* 50 120–127. 10.1016/j.appet.2007.06.007 17655972

[B88] Frederich R. C. Hamann A. Anderson S. Lollmann B. Lowell B. B. Flier J. S. (1995). Leptin levels reflect body lipid content in mice: evidence for diet-induced resistance to leptin action. *Nat. Med.* 1 1311–1314. 10.1038/nm1295-1311 7489415

[B89] Frost G. Sleeth M. L. Sahuri-Arisoylu M. Lizarbe B. Cerdan S. Brody L. (2014). The short-chain fatty acid acetate reduces appetite via a central homeostatic mechanism. *Nat. Commun.* 5:3611. 10.1038/ncomms4611 24781306 PMC4015327

[B90] Garbinsky E. N. Morewedge C. K. Shiv B. (2014). Does liking or wanting determine repeat consumption delay? *Appetite* 72 59–65. 10.1016/j.appet.2013.09.025 24104055

[B91] Garcia-Cabrerizo R. Carbia C. Kj O. R. Schellekens H. Cryan J. F. (2021). Microbiota-gut-brain axis as a regulator of reward processes. *J. Neurochem.* 157 1495–1524. 10.1111/jnc.15284 33368280

[B92] Gartner S. N. Aidney F. Klockars A. Prosser C. Carpenter E. A. Isgrove K. (2018). Intragastric preloads of l-tryptophan reduce ingestive behavior via oxytocinergic neural mechanisms in male mice. *Appetite* 125 278–286. 10.1016/j.appet.2018.02.015 29471071

[B93] Gibson G. R. Hutkins R. Sanders M. E. Prescott S. L. Reimer R. A. Salminen S. J. (2017). Expert consensus document: the International Scientific Association for Probiotics and Prebiotics (Isapp) consensus statement on the definition and scope of prebiotics. *Nat. Rev. Gastroenterol. Hepatol.* 14 491–502. 10.1038/nrgastro.2017.75 28611480

[B94] Goodlad R. A. Ratcliffe B. Fordham J. P. Ghatei M. A. Domin J. Bloom S. R. (1989). Plasma enteroglucagon, gastrin and peptide Yy in conventional and germ-free rats refed with a fibre-free or fibre-supplemented diet. *Q. J. Exp. Physiol.* 74 437–442. 10.1113/expphysiol.1989.sp003291 2552491

[B95] Griffioen-Roose S. Finlayson G. Mars M. Blundell J. E. De Graaf C. (2010). Measuring food reward and the transfer effect of sensory specific satiety. *Appetite* 55 648–655. 10.1016/j.appet.2010.09.018 20870002

[B96] Hamamah S. Aghazarian A. Nazaryan A. Hajnal A. Covasa M. (2022). Role of microbiota-gut-brain axis in regulating dopaminergic signaling. *Biomedicines* 10:436. 10.3390/biomedicines10020436 35203645 PMC8962300

[B97] Han W. Tellez L. A. Perkins M. H. Perez I. O. Qu T. Ferreira J. (2018a). A neural circuit for gut-induced reward. *Cell* 175 887–888. 10.1016/j.cell.2018.10.018 30340046

[B98] Han W. Tellez L. A. Perkins M. H. Perez I. O. Qu T. Ferreira J. (2018b). A Neural Circuit for Gut-Induced Reward. *Cell* 175 665–678.e23. 10.1016/j.cell.2018.08.049 30245012 PMC6195474

[B99] Hankir M. K. Seyfried F. Hintschich C. A. Diep T. A. Kleberg K. Kranz M. (2017). Gastric Bypass Surgery Recruits a Gut Ppar-alpha-Striatal D1R Pathway to Reduce Fat Appetite in Obese Rats. *Cell Metab.* 25 335–344. 10.1016/j.cmet.2016.12.006 28065827

[B100] Hansen C. F. Vrang N. Sangild P. T. Jelsing J. (2013). Novel insight into the distribution of L-cells in the rat intestinal tract. *Am. J. Transl. Res.* 5 347–358. 23634245 PMC3633977

[B101] Hansen K. B. Rosenkilde M. M. Knop F. K. Wellner N. Diep T. A. Rehfeld J. F. (2011). 2-Oleoyl glycerol is a Gpr119 agonist and signals Glp-1 release in humans. *J. Clin. Endocrinol. Metab.* 96 E1409–E1417. 10.1210/jc.2011-0647 21778222

[B102] Hayes M. R. Leichner T. M. Zhao S. Lee G. S. Chowansky A. Zimmer D. (2011). Intracellular signals mediating the food intake-suppressive effects of hindbrain glucagon-like peptide-1 receptor activation. *Cell Metab.* 13 320–330. 10.1016/j.cmet.2011.02.001 21356521 PMC3108145

[B103] He Z. Gao Y. Lieu L. Afrin S. Cao J. Michael N. J. (2019). Direct and indirect effects of liraglutide on hypothalamic Pomc and Npy/Agrp neurons - Implications for energy balance and glucose control. *Mol. Metab.* 28 120–134. 10.1016/j.molmet.2019.07.008 31446151 PMC6822260

[B104] Hill C. Guarner F. Reid G. Gibson G. R. Merenstein D. J. Pot B. (2014). Expert consensus document. The International Scientific Association for Probiotics and Prebiotics consensus statement on the scope and appropriate use of the term probiotic. *Nat. Rev. Gastroenterol. Hepatol.* 11 506–514. 10.1038/nrgastro.2014.66 24912386

[B105] Hokfelt T. Blacker D. Broberger C. Herrera-Marschitz M. Snyder G. Fisone G. (2002). Some aspects on the anatomy and function of central cholecystokinin systems. *Pharmacol. Toxicol.* 91 382–386. 10.1034/j.1600-0773.2002.910617.x 12688383

[B106] Hommel J. D. Trinko R. Sears R. M. Georgescu D. Liu Z. W. Gao X. B. (2006). Leptin receptor signaling in midbrain dopamine neurons regulates feeding. *Neuron* 51 801–810. 10.1016/j.neuron.2006.08.023 16982424

[B107] Jiang T. Soussignan R. Schaal B. Royet J. P. (2015). Reward for food odors: an fmri study of liking and wanting as a function of metabolic state and Bmi. *Soc. Cogn. Affect. Neurosci.* 10 561–568. 10.1093/scan/nsu086 24948157 PMC4381239

[B108] Joly-Amado A. Cansell C. Denis R. G. Delbes A. S. Castel J. Martinez S. (2014). The hypothalamic arcuate nucleus and the control of peripheral substrates. *Best Pract. Res. Clin. Endocrinol. Metab.* 28 725–737. 10.1016/j.beem.2014.03.003 25256767

[B109] Kaelberer M. M. Buchanan K. L. Klein M. E. Barth B. B. Montoya M. M. Shen X. (2018). A gut-brain neural circuit for nutrient sensory transduction. *Science* 361:eaat5236. 10.1126/science.aat5236 30237325 PMC6417812

[B110] Kamegai J. Tamura H. Shimizu T. Ishii S. Sugihara H. Wakabayashi I. (2000). Central effect of ghrelin, an endogenous growth hormone secretagogue, on hypothalamic peptide gene expression. *Endocrinology* 141 4797–4800. 10.1210/endo.141.12.7920 11108296

[B111] Kennedy G. C. (1953). The role of depot fat in the hypothalamic control of food intake in the rat. *Proc. R. Soc. Lond. B Biol. Sci.* 140 578–596. 10.1098/rspb.1953.0009 13027283

[B112] Kenny P. J. (2011). Common cellular and molecular mechanisms in obesity and drug addiction. *Nat. Rev. Neurosci.* 12 638–651. 10.1038/nrn3105 22011680

[B113] Koh A. De Vadder F. Kovatcheva-Datchary P. Backhed F. (2016). From Dietary Fiber to Host Physiology: short-Chain Fatty Acids as Key Bacterial Metabolites. *Cell* 165 1332–1345. 10.1016/j.cell.2016.05.041 27259147

[B114] Konanur V. R. Hsu T. M. Kanoski S. E. Hayes M. R. Roitman M. F. (2020). Phasic dopamine responses to a food-predictive cue are suppressed by the glucagon-like peptide-1 receptor agonist Exendin-4. *Physiol. Behav.* 215:112771. 10.1016/j.physbeh.2019.112771 31821815 PMC8112128

[B115] Kringelbach M. L. Stein A. Van Hartevelt T. J. (2012). The functional human neuroanatomy of food pleasure cycles. *Physiol. Behav.* 106 307–316. 10.1016/j.physbeh.2012.03.023 22487544

[B116] Lan H. Vassileva G. Corona A. Liu L. Baker H. Golovko A. (2009). Gpr119 is required for physiological regulation of glucagon-like peptide-1 secretion but not for metabolic homeostasis. *J. Endocrinol.* 201 219–230. 10.1677/JOE-08-0453 19282326

[B117] Latorre R. Sternini C. De Giorgio R. Greenwood-Van Meerveld B. (2016). Enteroendocrine cells: a review of their role in brain-gut communication. *Neurogastroenterol. Motil.* 28 620–630. 10.1111/nmo.12754 26691223 PMC4842178

[B118] Lau B. K. Cota D. Cristino L. Borgland S. L. (2017). Endocannabinoid modulation of homeostatic and non-homeostatic feeding circuits. *Neuropharmacology* 124 38–51. 10.1016/j.neuropharm.2017.05.033 28579186

[B119] Lauffer L. M. Iakoubov R. Brubaker P. L. (2009). Gpr119 is essential for oleoylethanolamide-induced glucagon-like peptide-1 secretion from the intestinal enteroendocrine L-cell. *Diabetes* 58 1058–1066. 10.2337/db08-1237 19208912 PMC2671052

[B120] Le Merrer J. Becker J. A. Befort K. Kieffer B. L. (2009). Reward processing by the opioid system in the brain. *Physiol. Rev.* 89 1379–1412. 10.1152/physrev.00005.2009 19789384 PMC4482114

[B121] le Roux C. W. Batterham R. L. Aylwin S. J. Patterson M. Borg C. M. Wynne K. J. (2006). Attenuated peptide Yy release in obese subjects is associated with reduced satiety. *Endocrinology* 147 3–8. 10.1210/en.2005-0972 16166213

[B122] Lebrun L. J. Lenaerts K. Kiers D. Pais De Barros J. P. Le Guern N. Plesnik J. (2017). Enteroendocrine L Cells Sense Lps after Gut Barrier Injury to Enhance Glp-1 Secretion. *Cell Rep.* 21 1160–1168. 10.1016/j.celrep.2017.10.008 29091756

[B123] Li J. Hou L. Wang C. Jia X. Qin X. Wu C. (2018). Short term intrarectal administration of sodium propionate induces antidepressant-like effects in rats exposed to chronic unpredictable mild stress. *Front. Psychiatry* 9:454. 10.3389/fpsyt.2018.00454 30319461 PMC6170646

[B124] Liddle R. A. (2019). Neuropods. *Cell. Mol. Gastroenterol. Hepatol.* 7 739–747. 10.1016/j.jcmgh.2019.01.006 30710726 PMC6463090

[B125] Liou A. P. Lu X. Sei Y. Zhao X. Pechhold S. Carrero R. J. (2011). The G-protein-coupled receptor Gpr40 directly mediates long-chain fatty acid-induced secretion of cholecystokinin. *Gastroenterology* 140 903–912. 10.1053/j.gastro.2010.10.012 20955703 PMC4717904

[B126] Lonnqvist F. Arner P. Nordfors L. Schalling M. (1995). Overexpression of the obese (ob) gene in adipose tissue of human obese subjects. *Nat. Med.* 1 950–953. 10.1038/nm0995-950 7585223

[B127] Lorenz D. N. Goldman S. A. (1982). Vagal mediation of the cholecystokinin satiety effect in rats. *Physiol. Behav.* 29 599–604. 10.1016/0031-9384(82)90226-8 6294698

[B128] Louis P. Flint H. J. (2016). Formation of propionate and butyrate by the human colonic microbiota. *Environ. Microbiol.* 19 29–41. 10.1111/1462-2920.13589 27928878

[B129] Manca C. Boubertakh B. Leblanc N. Deschenes T. Lacroix S. Martin C. (2020a). Germ-free mice exhibit profound gut microbiota-dependent alterations of intestinal endocannabinoidome signaling. *J. Lipid. Res.* 61 70–85. 10.1194/jlr.RA119000424 31690638 PMC6939599

[B130] Manca C. Shen M. Boubertakh B. Martin C. Flamand N. Silvestri C. (2020b). Alterations of brain endocannabinoidome signaling in germ-free mice. *Biochim. Biophys. Acta Mol. Cell Biol. Lipids* 1865:158786. 10.1016/j.bbalip.2020.158786 32795503

[B131] Mannucci E. Ognibene A. Cremasco F. Bardini G. Mencucci A. Pierazzuoli E. (2000). Glucagon-like peptide (Glp)-1 and leptin concentrations in obese patients with Type 2 diabetes mellitus. *Diabet. Med.* 17 713–719. 11110504 10.1046/j.1464-5491.2000.00367.x

[B132] Montague C. T. Farooqi I. S. Whitehead J. P. Soos M. A. Rau H. Wareham N. J. (1997). Congenital leptin deficiency is associated with severe early-onset obesity in humans. *Nature* 387 903–908. 9202122 10.1038/43185

[B133] Monteleone A. M. Piscitelli F. Dalle Grave R. El Ghoch M. Di Marzo V. Maj M. (2017). Peripheral Endocannabinoid Responses to Hedonic Eating in Binge-Eating Disorder. *Nutrients* 9:1377. 10.3390/nu9121377 29261146 PMC5748827

[B134] Morales M. Margolis E. B. (2017). Ventral tegmental area: cellular heterogeneity, connectivity and behaviour. *Nat. Rev. Neurosci.* 18 73–85. 28053327 10.1038/nrn.2016.165

[B135] Morley J. E. Levine A. S. Grace M. Kneip J. (1985). Peptide Yy (Pyy), a potent orexigenic agent. *Brain Res.* 341 200–203. 3840047 10.1016/0006-8993(85)91490-8

[B136] Mortensen K. Christensen L. L. Holst J. J. Orskov C. (2003). Glp-1 and Gip are colocalized in a subset of endocrine cells in the small intestine. *Regul. Pept.* 114 189–196. 10.1016/s0167-0115(03)00125-3 12832109

[B137] Morton G. J. Cummings D. E. Baskin D. G. Barsh G. S. Schwartz M. W. (2006). Central nervous system control of food intake and body weight. *Nature* 443 289–295. 16988703 10.1038/nature05026

[B138] Morton G. J. Meek T. H. Schwartz M. W. (2014). Neurobiology of food intake in health and disease. *Nat. Rev. Neurosci.* 15 367–378. 24840801 10.1038/nrn3745 PMC4076116

[B139] Moss C. E. Glass L. L. Diakogiannaki E. Pais R. Lenaghan C. Smith D. M. (2015). Lipid derivatives activate Gpr119 and trigger Glp-1 secretion in primary murine L-cells. *Peptides* 77 16–20. 10.1016/j.peptides.2015.06.012 26144594 PMC4788502

[B140] Murray S. Tulloch A. Gold M. S. Avena N. M. (2014). Hormonal and neural mechanisms of food reward, eating behaviour and obesity. *Nat. Rev. Endocrinol.* 10 540–552. 24958311 10.1038/nrendo.2014.91

[B141] Obici S. Feng Z. Karkanias G. Baskin D. G. Rossetti L. (2002). Decreasing hypothalamic insulin receptors causes hyperphagia and insulin resistance in rats. *Nat. Neurosci.* 5 566–572. 12021765 10.1038/nn0602-861

[B142] Olds J. Milner P. (1954). Positive reinforcement produced by electrical stimulation of septal area and other regions of rat brain. *J. Comp. Physiol. Psychol*. 47 419–427 10.1037/h0058775 13233369

[B143] Olds M. E. Fobes J. L. (1981). The central basis of motivatio: intracranial self-stimulation studies. *Annu. Rev. Physiol.* 32 523–574. 10.1146/annurev.ps.32.020181.002515 7015997

[B144] Page K. A. Seo D. Belfort-Deaguiar R. Lacadie C. Dzuira J. Naik S. (2011). Circulating glucose levels modulate neural control of desire for high-calorie foods in humans. *J. Clin. Invest.* 121 4161–4169. 10.1172/JCI57873 21926468 PMC3195474

[B145] Palmiter R. D. (2007). Is dopamine a physiologically relevant mediator of feeding behavior? *Trends Neurosci.* 30 375–381. 17604133 10.1016/j.tins.2007.06.004

[B146] Plovier H. Cani P. D. (2017). Enteroendocrine cells: metabolic relays between microbes and their host. *Endocr. Dev.* 32 139–164. 28898875 10.1159/000475736

[B147] Plovier H. Everard A. Druart C. Depommier C. Van Hul M. Geurts L. (2017). A purified membrane protein from *Akkermansia muciniphila* or the pasteurized bacterium improves metabolism in obese and diabetic mice. *Nat. Med.* 23 107–113. 27892954 10.1038/nm.4236

[B148] Polak J. M. Bloom S. R. Rayford P. L. Pearse A. G. Buchan A. M. Thompson J. C. (1975). Identification of cholecystokinin-secreting cells. *Lancet* 2 1016–1018. 53500 10.1016/s0140-6736(75)90297-4

[B149] Polk S. E. Schulte E. M. Furman C. R. Gearhardt A. N. (2017). Wanting and liking: separable components in problematic eating behavior? *Appetite* 115 45–53. 10.1016/j.appet.2016.11.015 27840087 PMC5796412

[B150] Poschel B. P. Ninteman FW. (1963). Norepinephrine: a possible excitatory Neurohormone of the reward system. *Life Sci.* 10 782–788. 10.1016/0024-3205(63)90087-0 14076601

[B151] Psichas A. Sleeth M. L. Murphy K. G. Brooks L. Bewick G. A. Hanyaloglu A. C. (2015). The short chain fatty acid propionate stimulates Glp-1 and Pyy secretion via free fatty acid receptor 2 in rodents. *Int. J. Obes.* 39 424–429. 10.1038/ijo.2014.153 25109781 PMC4356745

[B152] Ranganath L. R. Beety J. M. Morgan L. M. Wright J. W. Howland R. Marks V. (1996). Attenuated Glp-1 secretion in obesity: Cause or consequence? *Gut* 38 916–919. 8984033 10.1136/gut.38.6.916 PMC1383202

[B153] Regnier M. Van Hul M. Knauf C. Cani P. D. (2021). Gut microbiome, endocrine control of gut barrier function and metabolic diseases. *J. Endocrinol.* 248 R67–R82. 33295880 10.1530/JOE-20-0473

[B154] Reigstad C. S. Salmonson C. E. Rainey J. F. III Szurszewski J. H. Linden D. R. Sonnenburg J. L. (2015). Gut microbes promote colonic serotonin production through an effect of short-chain fatty acids on enterochromaffin cells. *FASEB J.* 29 1395–1403. 10.1096/fj.14-259598 25550456 PMC4396604

[B155] Rinaman L. (2010). Ascending projections from the caudal visceral nucleus of the solitary tract to brain regions involved in food intake and energy expenditure. *Brain Res.* 1350 18–34. 10.1016/j.brainres.2010.03.059 20353764 PMC2909454

[B156] Roberge J. N. Brubaker P. L. (1993). Regulation of intestinal proglucagon-derived peptide secretion by glucose-dependent insulinotropic peptide in a novel enteroendocrine loop. *Endocrinology* 133 233–240. 10.1210/endo.133.1.8319572 8319572

[B157] Robinson T. E. Berridge K. C. (2003). Addiction. *Annu. Rev. Psychol.* 54 25–53. 12185211 10.1146/annurev.psych.54.101601.145237

[B158] Robinson T. E. Berridge KC. (1993). The neural basis of drug craving: an incentive-sensitization theory of addiction. *Brain Res.* 18 247–291. 10.1016/0165-0173(93)90013-p 8401595

[B159] Ronveaux C. C. Tome D. Raybould H. E. (2015). Glucagon-like peptide 1 interacts with ghrelin and leptin to regulate glucose metabolism and food intake through vagal afferent neuron signaling. *J. Nutr.* 145 672–680. 10.3945/jn.114.206029 25833771 PMC4381768

[B160] Rosenstein D. Oster H. (1988). Differential Facial Responses to Four Basic Tastes in Newborns. *Child Dev.* 59 1555–1568. 3208567

[B161] Sclafani A. Ackroff K. Schwartz G. J. (2003). Selective effects of vagal deafferentation and celiac-superior mesenteric ganglionectomy on the reinforcing and satiating action of intestinal nutrients. *Physiol. Behav.* 78 285–294. 10.1016/s0031-9384(02)00968-x 12576127

[B162] Secher A. Jelsing J. Baquero A. F. Hecksher-Sorensen J. Cowley M. A. Dalboge L. S. (2014). The arcuate nucleus mediates Glp-1 receptor agonist liraglutide-dependent weight loss. *J. Clin. Invest.* 124 4473–4488. 10.1172/JCI75276 25202980 PMC4215190

[B163] Seo S. Ju S. Chung H. Lee D. Park S. (2008). Acute effects of glucagon-like peptide-1 on hypothalamic neuropeptide and Amp activated kinase expression in fasted rats. *Endocr. J.* 55 867–874. 10.1507/endocrj.k08e-091 18506089

[B164] Sorokowska A. Schoen K. Hummel C. Han P. Warr J. Hummel T. (2017). Food-related odors activate dopaminergic brain areas. *Front. Hum. Neurosci.* 11:625. 10.3389/fnhum.2017.00625 29311879 PMC5742189

[B165] Soto M. Herzog C. Pacheco J. A. Fujisaka S. Bullock K. Clish C. B. (2018). Gut microbiota modulate neurobehavior through changes in brain insulin sensitivity and metabolism. *Mol. Psychiatry* 23 2287–2301. 10.1038/s41380-018-0086-5 29910467 PMC6294739

[B166] Stice E. Figlewicz D. P. Gosnell B. A. Levine A. S. Pratt W. E. (2013). The contribution of brain reward circuits to the obesity epidemic. *Neurosci. Biobehav. Rev.* 37 2047–2058. 10.1016/j.neubiorev.2012.12.001 23237885 PMC3604128

[B167] Stice E. Spoor S. Bohon C. Veldhuizen M. G. Small D. M. (2008b). Relation of reward from food intake and anticipated food intake to obesity: a functional magnetic resonance imaging study. *J. Abnorm. Psychol.* 117 924–935. 19025237 10.1037/a0013600 PMC2681092

[B168] Stice E. Spoor S. Bohon C. Small D. M. (2008a). Relation between obesity and blunted striatal response to food is moderated by Taqia A1 allele. *Science* 322 449–452. 10.1126/science.1161550 18927395 PMC2681095

[B169] Stice E. Yokum S. Bohon C. Marti N. Smolen A. (2010). Reward circuitry responsivity to food predicts future increases in body mass: moderating effects of Drd2 and Drd4. *Neuroimage* 50 1618–1625. 20116437 10.1016/j.neuroimage.2010.01.081 PMC3987805

[B170] Strandwitz P. (2018). Neurotransmitter modulation by the gut microbiota. *Brain Res.* 1693 128–133. 29903615 10.1016/j.brainres.2018.03.015 PMC6005194

[B171] Sun X. Luquet S. Small D. M. (2017). Drd2: bridging the Genome and Ingestive Behavior. *Trends Cogn. Sci.* 21 372–384. 28372879 10.1016/j.tics.2017.03.004 PMC5745142

[B172] Swartz T. D. Duca F. A. De Wouters T. Sakar Y. Covasa M. (2012). Up-regulation of intestinal type 1 taste receptor 3 and sodium glucose luminal transporter-1 expression and increased sucrose intake in mice lacking gut microbiota. *Br. J. Nutr.* 107 621–630. 10.1017/S0007114511003412 21781379

[B173] Tan H. E. Sisti A. C. Jin H. Vignovich M. Villavicencio M. Tsang K. S. (2020). The gut-brain axis mediates sugar preference. *Nature* 580 511–516. 32322067 10.1038/s41586-020-2199-7 PMC7185044

[B174] Tellez L. A. Medina S. Han W. Ferreira J. G. Licona-Limon P. Ren X. (2013). A gut lipid messenger links excess dietary fat to dopamine deficiency. *Science* 341 800–802. 10.1126/science.1239275 23950538

[B175] Tennoune N. Chan P. Breton J. Legrand R. Chabane Y. N. Akkermann K. (2014). Bacterial ClpB heat-shock protein, an antigen-mimetic of the anorexigenic peptide alpha-Msh, at the origin of eating disorders. *Transl. Psychiatry* 4:e458. 10.1038/tp.2014.98 25290265 PMC4350527

[B176] Thaler J. P. Guyenet S. J. Dorfman M. D. Wisse B. E. Schwartz M. W. (2013). Hypothalamic inflammation: marker or mechanism of obesity pathogenesis? *Diabetes* 62 2629–2634. 23881189 10.2337/db12-1605 PMC3717869

[B177] Thomsen K. R. (2015). Measuring anhedonia: impaired ability to pursue, experience, and learn about reward. *Front. Psychol.* 6:1409. 10.3389/fpsyg.2015.01409 26441781 PMC4585007

[B178] Thornton J. E. Cheung C. C. Clifton D. K. Steiner R. A. (1997). Regulation of hypothalamic proopiomelanocortin mrna by leptin in ob/ob mice. *Endocrinology* 138 5063–5066. 9348241 10.1210/endo.138.11.5651

[B179] Tracy A. L. Wee C. J. Hazeltine G. E. Carter R. A. (2015). Characterization of attenuated food motivation in high-fat diet-induced obesity: critical roles for time on diet and reinforcer familiarity. *Physiol. Behav.* 141 69–77. 10.1016/j.physbeh.2015.01.008 25582517

[B180] Trapp S. Brierley D. I. (2022). Brain Glp-1 and the regulation of food intake: Glp-1 action in the brain and its implications for Glp-1 receptor agonists in obesity treatment. *Br. J. Pharmacol.* 179 557–570. 10.1111/bph.15638 34323288 PMC8820179

[B181] Tschop M. Smiley D. L. Heiman M. L. (2000). Ghrelin induces adiposity in rodents. *Nature* 407 908–913. 11057670 10.1038/35038090

[B182] van de Wouw M. Boehme M. Lyte J. M. Wiley N. Strain C. O’sullivan O. (2018). Short-chain fatty acids: microbial metabolites that alleviate stress-induced brain-gut axis alterations. *J. Physiol.* 596 4923–4944. 10.1113/JP276431 30066368 PMC6187046

[B183] van de Wouw M. Schellekens H. Dinan T. G. Cryan J. F. (2017). Microbiota-Gut-Brain Axis: modulator of Host Metabolism and Appetite. *J. Nutr.* 147 727–745. 28356427 10.3945/jn.116.240481

[B184] van der Klaauw A. A. Farooqi I. S. (2015). The hunger genes: pathways to obesity. *Cell* 161 119–132. 25815990 10.1016/j.cell.2015.03.008

[B185] Varela L. Horvath T. L. (2012). Leptin and insulin pathways in Pomc and Agrp neurons that modulate energy balance and glucose homeostasis. *Embo Rep.* 13 1079–1086. 23146889 10.1038/embor.2012.174 PMC3512417

[B186] Vazquez E. Barranco A. Ramirez M. Gruart A. Delgado-Garcia J. M. Jimenez M. L. (2016). Dietary 2’-Fucosyllactose Enhances Operant Conditioning and Long-Term Potentiation via Gut-Brain Communication through the Vagus Nerve in Rodents. *PLoS One* 11:e0166070. 10.1371/journal.pone.0166070 27851789 PMC5113009

[B187] Verdich C. Toubro S. Buemann B. Lysgard Madsen J. Juul Holst J. Astrup A. (2001). The role of postprandial releases of insulin and incretin hormones in meal-induced satiety–effect of obesity and weight reduction. *Int. J. Obes. Relat. Metab. Disord.* 25 1206–1214. 10.1038/sj.ijo.0801655 11477506

[B188] Vijay-Kumar M. Aitken J. D. Carvalho F. A. Cullender T. C. Mwangi S. Srinivasan S. (2010). Metabolic syndrome and altered gut microbiota in mice lacking Toll-like receptor 5. *Science* 328 228–231. 20203013 10.1126/science.1179721 PMC4714868

[B189] Vilsboll T. Krarup T. Deacon C. F. Madsbad S. Holst J. J. (2001). Reduced postprandial concentrations of intact biologically active glucagon-like peptide 1 in type 2 diabetic patients. *Diabetes* 50 609–613. 10.2337/diabetes.50.3.609 11246881

[B190] Volkow N. D. Wang G. J. Baler R. D. (2011). Reward, dopamine and the control of food intake: implications for obesity. *Trends Cogn. Sci.* 15 37–46. 21109477 10.1016/j.tics.2010.11.001 PMC3124340

[B191] Vucetic Z. Kimmel J. Reyes T. M. (2011). Chronic high-fat diet drives postnatal epigenetic regulation of mu-opioid receptor in the brain. *Neuropsychopharmacology* 36 1199–1206. 10.1038/npp.2011.4 21326195 PMC3077442

[B192] Wang G. J. Volkow N. D. Logan J. Pappas N. R. Wong C. T. Zhu W. (2001). Brain dopamine and obesity. *Lancet* 357 354–357. 11210998 10.1016/s0140-6736(00)03643-6

[B193] Wang X. F. Liu J. J. Xia J. Liu J. Mirabella V. Pang Z. P. (2015). Endogenous Glucagon-like Peptide-1 Suppresses High-Fat Food Intake by Reducing Synaptic Drive onto Mesolimbic Dopamine Neurons. *Cell Rep.* 12 726–733. 10.1016/j.celrep.2015.06.062 26212334 PMC4860285

[B194] Wang Y. B. De Lartigue G. Page A. J. (2020). Dissecting the Role of Subtypes of Gastrointestinal Vagal Afferents. *Front. Physiol.* 11:643. 10.3389/fphys.2020.00643 PMC7300233 32595525

[B195] Wang Y. Chandra R. Samsa L. A. Gooch B. Fee B. E. Cook J. M. (2011). Amino acids stimulate cholecystokinin release through the Ca2+-sensing receptor. *Am. J. Physiol. Gastrointest. Liver. Physiol.* 300 G528–G537. 21183662 10.1152/ajpgi.00387.2010 PMC3074989

[B196] Warrilow A. Turner M. Naumovski N. Somerset S. (2022). Role of cholecystokinin in satiation: a systematic review and meta-analysis. *Br. J. Nutr.* 14 1–25. 10.1017/S0007114522000381 35152916

[B197] Weaver G. A. Krause J. A. Miller T. L. Wolin M. J. (1988). Short chain fatty acid distributions of enema samples from a sigmoidoscopy population: an association of high acetate and low butyrate ratios with adenomatous polyps and colon cancer. *Gut* 29 1539–1543. 10.1136/gut.29.11.1539 3209110 PMC1433834

[B198] Wichmann A. Allahyar A. Greiner T. U. Plovier H. Lunden G. O. Larsson T. (2013). Microbial modulation of energy availability in the colon regulates intestinal transit. *Cell Host Microbe* 14 582–590. 10.1016/j.chom.2013.09.012 24237703

[B199] Willesen M. G. Kristensen P. Romer J. (1999). Co-localization of growth hormone secretagogue receptor and Npy mrna in the arcuate nucleus of the rat. *Neuroendocrinology* 70 306–316. 10.1159/000054491 10567856

[B200] Wilson B. D. Bagnol D. Kaelin C. B. Ollmann M. M. Gantz I. Watson S. J. (1999). Physiological and anatomical circuitry between Agouti-related protein and leptin signaling. *Endocrinology* 140 2387–2397. 10.1210/endo.140.5.6728 10218993

[B201] Wise R. A. (1977). Catecholamine theories of reward: a critical review. *Brain Res.* 152 215–247. 10.1016/0006-8993(78)90253-6 354753

[B202] Wise R. A. Rompre P. P. (1989). Brain dopamine and reward. *Annu. Rev. Psychol.* 40 191–225. 2648975 10.1146/annurev.ps.40.020189.001203

[B203] Wiss D. A. Avena N. Rada P. (2018). Sugar Addiction: from Evolution to Revolution. *Front. Psychiatry* 9:545. 10.3389/fpsyt.2018.00545 30464748 PMC6234835

[B204] Wren A. M. Seal L. J. Cohen M. A. Brynes A. E. Frost G. S. Murphy K. G. (2001). Ghrelin enhances appetite and increases food intake in humans. *J. Clin. Endocrinol. Metab.* 86 5992–5995. 11739476 10.1210/jcem.86.12.8111

[B205] Yang J. W. Kim H. S. Choi Y. W. Kim Y. M. Kang K. W. (2018). Therapeutic application of Gpr119 ligands in metabolic disorders. *Diabetes Obes. Metab.* 20 257–269. 28722242 10.1111/dom.13062

[B206] Ye L Liddle R. A. (2017). Gastrointestinal hormones and the gut connectome. *Curr. Opin. Endocrinol. Diabetes Obes.* 24 9–14. 10.1097/MED.0000000000000299 27820704 PMC5815400

[B207] Yoon H. S. Cho C. H. Yun M. S. Jang S. J. You H. J. Kim J. H. (2021). *Akkermansia muciniphila* secretes a glucagon-like peptide-1-inducing protein that improves glucose homeostasis and ameliorates metabolic disease in mice. *Nat. Microbiol.* 6 563–573. 10.1038/s41564-021-00880-5 33820962

[B208] Zhang Y. Proenca R. Maffei M. Barone M. Leopold L. Friedman J. M. (1994). Positional cloning of the mouse obese gene and its human homologue. *Nature* 372 425–432. 7984236 10.1038/372425a0

[B209] Zhou J. Martin R. J. Tulley R. T. Raggio A. M. Mccutcheon K. L. Shen L. (2008). Dietary resistant starch upregulates total Glp-1 and Pyy in a sustained day-long manner through fermentation in rodents. *Am. J. Physiol. Endocrinol. Metab.* 295 E1160–E1166. 10.1152/ajpendo.90637.2008 18796545 PMC2584810

